# Bacterial Lymphatic Metastasis in Infection and Immunity

**DOI:** 10.3390/cells11010033

**Published:** 2021-12-23

**Authors:** Matthew K. Siggins, Shiranee Sriskandan

**Affiliations:** 1 National Heart and Lung Institute, Imperial College London, London W2 1PG, UK; 2 Department of Infectious Disease, Imperial College London, London W12 0NN, UK; 3 MRC Centre for Molecular Bacteriology and Infection, Imperial College London, London SW7 2DD, UK

**Keywords:** bacteria, bacteraemia, bacterial dissemination, immunity, infection, invasion, lymphatics, lymphatic system, lymphatic metastasis, lymph nodes

## Abstract

Lymphatic vessels permeate tissues around the body, returning fluid from interstitial spaces back to the blood after passage through the lymph nodes, which are important sites for adaptive responses to all types of pathogens. Involvement of the lymphatics in the pathogenesis of bacterial infections is not well studied. Despite offering an obvious conduit for pathogen spread, the lymphatic system has long been regarded to bar the onward progression of most bacteria. There is little direct data on live virulent bacteria, instead understanding is largely inferred from studies investigating immune responses to viruses or antigens in lymph nodes. Recently, we have demonstrated that extracellular bacterial lymphatic metastasis of virulent strains of *Streptococcus pyogenes* drives systemic infection. Accordingly, it is timely to reconsider the role of lymph nodes as absolute barriers to bacterial dissemination in the lymphatics. Here, we summarise the routes and mechanisms by which an increasing variety of bacteria are acknowledged to transit through the lymphatic system, including those that do not necessarily require internalisation by host cells. We discuss the anatomy of the lymphatics and other factors that influence bacterial dissemination, as well as the consequences of underappreciated bacterial lymphatic metastasis on disease and immunity.

## 1. The Dogma of Bacterial Invasion

How a pathogen disseminates through its host is a critical question in understanding infection and immunity which has applications in the prevention, diagnosis, and treatment of infectious disease. Currently, thorough knowledge of the routes and mechanisms by which bacteria disseminate, and the relative contribution of different pathways to the development of serious infections and immune responses is lacking. Despite this, severe bloodstream infection and serious necrotizing infections are commonly believed to arise after invasion of the blood vessel endothelium by bacteria that have invaded deeper tissue from a superficial origin [1,2,3,4]. Conversely, while dissemination via the lymphatic system is sometimes mooted as a potential pathway that contributes to deeper invasion of microbes, it is usually considered to be insignificant due to inefficient uptake in tissues and effective filtration by lymph nodes [5,6,7,8]. As such, reference to the lymphatics in bacterial infection literature is often sparse and imprecise. Instead, a research focus on direct microbial disruption of epithelial and vascular endothelial cell layers dominates the perspectives on invasion [3,9,10]. Recently, a preponderance of studies demonstrating intracellular survival as a mechanism for bacterial pathogenesis has further reinforced the dogma of blood vessel invasion in bloodstream infection [11].

Here, we discuss concepts in bacterial invasion, outline the anatomy underlying paths of dissemination for invading bacteria, and consider the ability of lymph nodes to control metastatic bacteria and their impact on infection outcome. We present evidence to suggest that bacterial lymphatic metastasis and host–bacterial interactions within the lymph nodes are of greater prominence, in both invasion and development of immune responses, than previously considered.

## 2. Barriers to Studying Bacteria in the Lymphatics

Precisely tracking the dissemination of microbes is difficult, often requiring specialised surgery and advanced microscopy, with biosafety requirements presenting additional hurdles. As a result, the topic remains understudied.

The lymphatic system comprises an extensive, tissue-permeating network of vessels that returns fluid into the blood circulation via filtering lymph nodes. Until relatively recently, study of the lymphatics has been limited by a lack of specific markers [12,13,14]. As a result, investigation into the role of the lymphatic system in bacterial dissemination has been particularly neglected compared to other routes of invasion.

Lymphatic metastasis refers to the spread of a pathogenic agent from an initial to a secondary site via the lymphatics. Despite anatomical and historical data supporting such spread, only a handful of specialised intracellular lymph-tropic bacterial pathogens are widely considered to exploit the lymphatic system in this way during infection. For instance, *Mycobacterium bovis*, *Salmonella* spp., and *Yersinia pestis* are all believed to be ferried through the lymphatics to lymph nodes inside phagocytic cells, including dendritic cells, monocytes, and neutrophils [15,16,17].

However, data on virulent strains of other bacterial pathogens are lacking. Much current understanding is largely reliant on extrapolation from experiments with viruses, or killed or avirulent bacterial strains, which are likely to differ in behaviour to live virulent bacteria. As a result, prominent work describing effective mechanisms of viral capture and immune defence by lymph nodes have shaped perception and further sidelined the perceived importance of bacterial lymphatic dissemination in infection [18,19,20].

## 3. Bacteria in the Lymphatics

**Figure 1:**
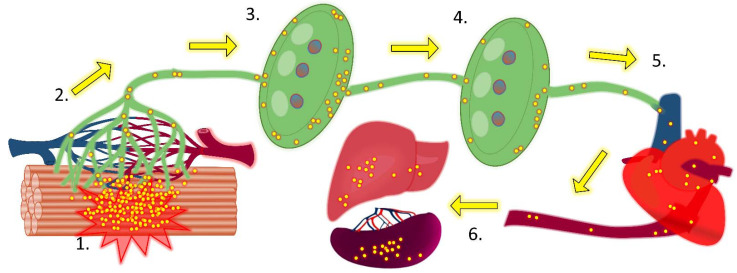
**Path of bacterial dissemination following intramuscular inoculation of bacteria**. **1.** Bacteria inoculated into tissue (here streptococcal intramuscular infection of a mouse is depicted, but similar results are obtained using other infection routes and models) preferentially and passively enter the initial lymphatics (green channels) rather than blood vessels (dark red and blue channels). **2.** Bacteria transit extracellularly along the lymphatics into collecting lymphatics and then enter local draining lymph nodes in afferent lymph. **3.** Bacteria accumulate in the subcapsular sinus and, particularly, the medullary sinuses of the lymph node. However, numerous bacteria escape the filtering activity of the lymph node and exit through the efferent lymphatic. **4.** Bacteria transit in efferent lymphatics to reach distant sequential draining lymph nodes and again accumulate in the sinuses. Although bacteria are initially present in lower numbers than in local draining lymph nodes, virulent strains can rapidly replicate within the lymph node niche, despite the activity of resident and recruited phagocytes and other leucocytes. **5.** A number of bacteria leave the lymph node through the efferent lymphatic vessel and drain with efferent lymph into collecting ducts before entering the bloodstream via the subclavian veins. **6.** Bacteria that have entered the bloodstream can now seed any tissue or other organs in the body. Many of these bacteria accumulate in the spleen and liver, and these organs play important roles in the clearance of bacteraemia.

We recently reported that extracellular bacterial lymphatic metastasis drives systemic infection by hypervirulent *emm*1 *Streptococcus* *pyogenes* strains which are associated with invasive human disease [21,22] (Figure 1). In these experiments, immune responses in the lymph node failed to abrogate onward extracellular passage of bacteria through the lymphatics [21]. We have also presented data that is consistent with a major role for lymphatic dissemination in infections with strains of *Pseudomonas aeruginosa* and *Klebsiella pneumoniae* [23]. As will be discussed, the virulence mechanisms important in driving lymphatic dissemination are shared by many bacterial species, suggesting that extracellular lymphatic dissemination is also important in other bacterial infections [21,22,23]. 

This evidence builds on sporadic studies, over the course of 150 years, which have reported the recovery of viable bacteria from local draining lymph nodes of various animals following infection with diverse bacterial species via a variety of inoculation routes (Table 1) [15,16,21,22,23,24,25,26,27,28,29,30,31,32,33,34,35,36]. There is considerably less data on bacteria in lymphatic sites beyond the initial local draining lymph nodes, such as in efferent lymphatics and lymph (Table 2) [15,21,23,37,38], and only a handful of studies have rigorously addressed the mechanisms of bacterial lymphatic spread [15,16,21,38]. As a result, much remains unknown about the initial dissemination to draining lymph nodes and transit beyond to efferent lymphatics, lymph, and systemic circulation for many important bacterial pathogens.

## 4. Extracellular Transit of Intracellular Bacteria

While this review focuses on the extracellular paths of lymphatic dissemination, it is important to address the dogma concerning infections with facultative intracellular lymph-tropic bacterial pathogens, such as *Salmonella* and *Y. pestis*. These infections are conventionally considered to be driven by bacteria transiting intracellularly in phagocytic cells that permit replication and function as “trojan horses” [15,16,17]. However, substantial data exists to suggest that extracellular lymphatic metastasis is important in pathogenesis [16,25,26,28,38,41].

It is clear that phagocytic cells, including dendritic cells, monocytes, and neutrophils, can carry bacteria to lymph nodes, but prominent extracellular lymphatic dissemination occurs during infections with *Salmonella* and *Y. pestis* [16,25,26,30,38]. Researchers who have adequately assessed lymphatics and lymph for extracellular bacteria have overwhelmingly found free bacteria in higher numbers than cell-associated bacteria [16,25,26,30,38]. Such high proportions of extracellular bacteria in the lymphatics have been suggested to be an artefact of large challenge doses; however, free bacteria are the most populous form in lymph following low dose inoculation of *Y. pestis* (similar to that delivered in a flea bite) [26]. Free bacteria are also still present in lymph 24 hours after infection with *Salmonella* [16], demonstrating that the phenomenon is not due to an initial overspill of bacteria.

There is evidence indicating that extracellular free bacteria in lymph are more important in subsequent invasion than cell-associated bacteria. Through analysis of *Salmonella* type III secretion system 1 and 2 mutants, Pullinger and colleagues found that replication at the initial site of infection, but not intracellular replication, was required for early lymphatic translocation of bacteria [38]. Additionally, in *Y. pestis* infection, free movement of bacteria into draining lymph nodes precedes recruitment and transit of phagocytes [26,42,43]. Furthermore, throughout early infection of the lymph node, *Y. pestis* remain mainly extracellular, with initial bacterial expansion occurring in the periphery (where particles from afferent lymph arrive), rather than in the more central areas (where most trafficking phagocytes principally enter, or home towards) [42].

Cell-associated bacteria in lymph are typically assumed to be internalised. However, often cellular location is not rigorously established; frequently, only gentamicin assays are employed to verify that bacteria are intracellular. While such assays are helpful in determining the cellular positioning of bacteria, the “black box” element means that they do not amount to definitive analysis. In contrast, imaging of cells in lymph appears to show *Salmonella* associated with cellular surfaces, rather than exclusively internalised [16]. Interestingly, the extracellular pathogen *S. pyogenes* has been found to associate with the surfaces of lymphocytes and other cells in efferent lymph through unknown means [21]. Many lymphatic observations and dissemination kinetics recorded for (classically considered) intracellular bacteria are strikingly similar to those obtained with (classically considered) extracellular bacteria [21]. Based on current evidence, it is far from conclusive that intracellular bacteria drive lymphatic spread and invasion of *Salmonella*, *Y. pestis* and other similar pathogens. The situation is likely more complex and further research of lymphatic dissemination and host-cell interaction is needed.

## 5. Role of Virulence in Bacterial Dissemination

In naming examples of specific bacterial species associated with lymphatic spread, it is prudent at this point to highlight that significant differences in infection exist not just between bacterial species, but between strains of the same species. At the broadest level, highly virulent pathogenic bacterial strains can subvert immune mechanisms that readily control less virulent strains, resulting in differing host cell–pathogen interactions and greatly contrasting invasion outcomes [44,45,46]. More specifically, differences can be dependent on even a single virulence factor, as for the impact of exopolysaccharides in dissemination [21,23,47]. Hence, it is important to determine host–pathogen interactions using characterised virulent clinical isolates, as opposed to classical laboratory strains which sometimes possess limited virulence, or else have distinct pathogenicity to contemporary circulating strains.

The selection of appropriate virulent strains of bacteria for study is further complicated by differences in susceptibility between humans and chosen animal models, as well as tissue tropism. For example, although *Staphylococcus aureus* USA300 (which has been the focus of several prominent studies of lymphatic interactions with bacteria using mouse models) is considered an invasive human pathogen, it displays poor virulence in mice beyond the local site of infection [48]. Unfortunately, strain selection is a particularly relevant issue in the investigation of lymphatic spread, as the low throughput methods frequently required, such as invasive microscopy, and immunological focus of much of the work mean that often only a small selection, or even just one strain, of bacteria are studied.

## 6. Importance of Bacterial Dissemination

The genitourinary tract, respiratory tract, and skin are among the most common initial infection sites that lead to secondary bacteremia [49]. Following adhesion and colonisation, pathogenic bacteria can propagate and invade underlying tissues, facilitated by the production of tissue-degrading enzymes, toxins, effector proteins, and a compliant weakened host immune response [9,50,51]. In these cases, secondary bacteremia can result due to seeding from the established primary infection site. While bacteremia is often secondary, it can also present as a primary infection that lacks an identifiable initial site of infection.

The presence of bacteria in the bloodstream is not a requirement for a bacterial infection to become life-threatening but it can be a critical step. Bloodstream infections contribute to a dysregulated inflammatory response and the development of sepsis, as well as facilitating hematogenous seeding of metastatic infection foci, as commonly suspected in necrotizing fasciitis and myositis [52,53]. Murine models show that death is associated with systemic dissemination of bacteria rather than the bacterial load at an initial infection site [21,54,55]. Furthermore, among patients with sepsis resulting from bacterial infection, higher mortality is observed in those who are bacteraemic [56].

The ability of the spleen and liver to capture and destroy bacteria present in the bloodstream, largely through the activity of macrophages and neutrophils, is well established [29,57,58,59,60,61]. This activity is usually adequate to contain mild and transient bacteraemia that may result from dental procedures, operations (such as tonsillectomy), or infection [62,63]. However, in susceptible individuals, sufficiently virulent bacteria can sometimes subvert these immune defences and multiply within the bloodstream or intravascular foci to drive serious infection [64,65].

Lymphatic dissemination from tissue gives access to lymph nodes and subsequently the blood. The intralymphatic metastatic infection foci that seed bacteraemia, previously acknowledged as important in diseases caused by *Bacillus anthracis* and *Y. pestis*, have recently been identified in *S. pyogenes* infection, and may be important in driving infection with other bacterial species [21,24,26,40].

Beyond invasive disease, as lymph nodes are critical in orchestrating robust adaptive immunity against bacteria, lymphatic dissemination has significant potential to impact immune responses. The presence of *S. aureus* and accompanying inflammation has been demonstrated to disrupt the development of immunity and the *Salmonella* bacterial burden is associated with delays in the formation of germinal centres [66,67,68]. Furthermore, bacterial immunostimulatory molecules have been measured in draining lymph nodes following staphylococcal soft tissue infection [69]. This is discussed in further detail in Section 21, Impact of Bacteria in Lymph Nodes on Immunity.

## 7. Challenging the Dogma of Bacterial Invasion

Despite the ability of some bacteria to invade blood endothelial cells, the anatomy of the vasculature dictates that lymphatic dissemination offers the path of least resistance to reach the bloodstream and should be considered the most logical default route. Review of data from animal models largely supports this interpretation, with the rapid appearance of bacteria in draining lymph nodes or efferent lymph prior to detection in blood consistently reported [21,23,37]. While these studies demonstrate a close association of bacterial load in lymph and blood at later timepoints, tellingly, the quantities of bacteria in blood lag those in lymph, indicating that direct bacterial entry into the bloodstream does not become dominant later in infection. Furthermore, enhanced bacterial dissemination to the bloodstream by both commensals and pathogens is observed following removal of draining lymph nodes, demonstrating that bacteria transit along lymphatic vessels [8,70]. Collectively, these observations show that many invasive bacterial infection animal models are driven by lymphatic dissemination.

In human disease too, many infections have clinical evidence supportive of the lymphatic involvement in dissemination (Table 3). Upwards of a quarter of bacterial bloodstream infections lack a known focus of infection [49,71,72]. This clinical presentation is inconsistent with the prevailing concept that bacteraemia results following significant local infection and subsequent direct invasion of blood vessels. These cryptic infections, which make up a substantial proportion of total severe infections for some important bacterial pathogens, lack any obvious portal of entry other than the known mucosal sites where the pathogen can be identified.

*S. pyogenes* has particularly firm associations with lymphatic pathology, and up to a quarter of invasive *S. pyogenes* infections present as bloodstream infection without any obvious infection source [73,74]. Invasive infections of deeper tissues, such as the fascia and muscle, often also present without signs of peripheral infection: almost half of invasive streptococcal necrotising soft tissue infections lack any clear portal of entry [75,76,77]. Other bacteria, including *Staphylococcus* spp., are also detected in lymph node biopsies of patients with lymphadenitis [78]. Furthermore, bacteria not traditionally associated with lymphangitic spread, including Gram negative *Escherichia* and *Klebsiella* species, have been recovered from lymph nodes in patients undergoing surgery [79,80,81]. There is some evidence that lymphatics may also serve as a route of bacterial dissemination for these bacteria despite the lack of a strong link to lymphatic pathology [29,82].

It should be noted that lymphatic pathologies are most identified in milder, localised infections, such as streptococcal erysipelas and cellulitis, and are particularly prevalent in individuals with impaired lymphatic drainage [83]. This suggests that it is the extended association of bacteria with local lymphatics of superficial tissues that results in lymphatic pathology, rather than transient passage along lymphatic vessels. Hence, a lack of lymphatic inflammation does not preclude lymphatic dissemination. Accordingly, lymphatic dissemination of a bacterial species should not be ruled out based on an apparent lack of lymphatic pathology.

Absence of a clear infection source in seemingly cryptic infections removes the diagnostic clues, leading to worse patient outcomes [84], coupled with an inability to achieve “source control” when treating a septic patient. While identification of a focus of infection can be challenging, and the failure to detect a primary infection site does not preclude its existence, bloodstream infections that arise without an obvious primary infection site provide persuasive evidence for the significance of lymphatic routes of invasion.

## 8. Initial Events in Infection

Surfaces in contact with the external environment, such as the skin and mucosa, are constantly exposed to bacteria. Accordingly, these sites have multiple layers of protection against infection; chemical, physical, and biological defenses must be bypassed before a potential pathogen is able to contact the epithelium. Epithelial cells themselves form tight cell-to-cell junctions which function as a barrier that excludes the passage of bacteria, though pathogens may breach the epithelium following antecedent injury or microbial-mediated disruption of cell layers [51,85,86].

**Figure 2:**
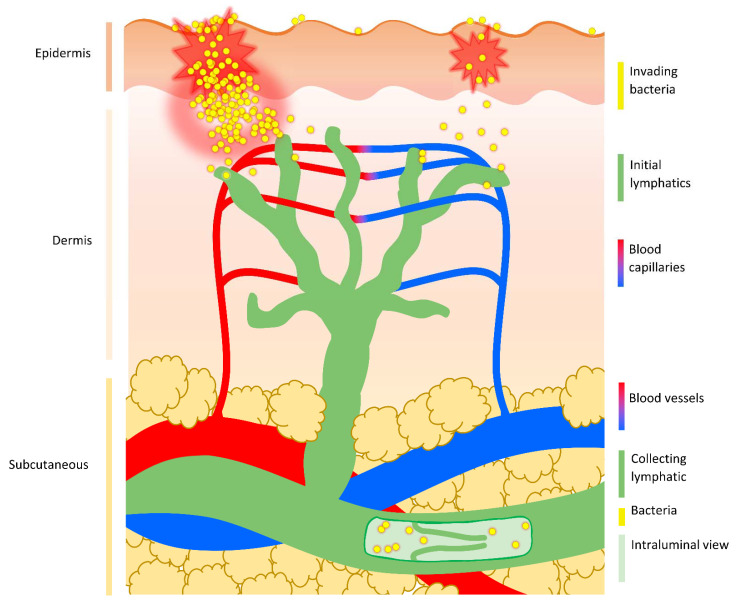
**Route of bacterial dissemination from the skin.** Following damage to the superficial epidermis of the skin, bacteria can either multiply at the peripheral site generating inflammation and promoting tissue damage, or passively move through the breached barrier and into underlying tissue without creating noticeable signs of infection at the site of entry (as hypothesised in cryptic infections). While the epidermis is devoid of any vasculature, blood capillaries and initial lymphatic vessels permeate the dermis. Bacteria are preferentially taken up by dermal lymphatic vessels and transported passively with lymph flow into deeper-lying collecting lymphatics. These larger lymphatic vessels run through the subcutaneous layer, possess secondary valves that prevent backflow, and carry lymph to draining lymph nodes.

Upon damage to the epithelium, host recognition of damage- and pathogen/microbial-associated molecular patterns (D-/PAMPs) by pattern recognition receptors (PRRs) triggers signaling cascades that drive induction of inflammation [87]. Expression of proinflammatory cytokines, activation of in situ immune cells, and rapid recruitment of additional immune cells create a highly antimicrobial environment that combats microbial replication and invasion of the underlying tissues. The interstitium, which lies below the epithelium, contains blood capillaries and initial lymphatics. Both vessels provide a potential portal into deeper tissue and the systemic blood circulation, thus permitting escape from the focused immune response at the site of initial infection in superficial tissues (Figure 2).

## 9. Anatomy of Bacterial Invasion I: Vessels and Fluid Flow in the Interstitium

Bacteria that breach the epithelial layer enter the interstitium. This interstitial space contains the extracellular matrix (ECM), as well as blood capillaries and initial lymphatics. Fluid flow in this compartment is essential to sustain cells and can also carry particles from the tissue into systemic circulation. Many bacteria that cause systemic infections lack a means of propulsion and so their movement is reliant on fluid flow within the body.

Fluid enters the interstitial space from the arterial end of the capillaries due to hydrostatic pressure and interstitial fluid is removed via the initial lymphatics, as well as through venous capillaries [88]. Small sized particles (≤10 nm diameter) introduced into the interstitium by injection (including intramuscular, intradermal, and subcutaneous routes) diffuse rapidly and are preferentially carried away by blood capillaries [89].

Capillaries are ~10 µm in diameter but, in the majority of tissues, transcapillary passage of particles bigger than 10 nm is prevented by tight endothelial cell junctions and complete basement membranes [90]: the basement membrane is a thin but dense sheet of specialised ECM that borders the basal side of the epithelial and endothelial cell layers and can restrict the movement of molecules. As spherical (cocci) bacteria have an average diameter of 0.5–2 µm and rod-shaped (bacilli) bacteria are typically around 0.5–1 µm wide and 1–4 µm long, bacteria cannot passively enter vascular capillaries in most tissues. Bacteria can fit through the larger gaps between the endothelial cell junctions of the specialised sinusoidal capillaries of the spleen and liver, but the deep proximal position of these organs means that sinusoidal blood capillaries are not relevant in initial bacterial entry into the bloodstream.

Initial lymphatics are more permeable than blood capillaries and can accommodate entry of much larger particles than blood capillaries. Bacteria can be observed in the initial lymphatics in both experimental animal models and during bacterial human disease. For example, *S. pyogenes* and *Y. pestis* are present extracellularly in local initial lymphatics following intradermal injection of mice [22,26], and bacteria have been described in the lymphatic vessels of puerperal sepsis patients, dating back to the early 20th century [91,92]. Moreover, evidence of bacterial presence in the lymphatics, in the form of lymphangitis and lymphadenitis, is commonly observed in bacterial infections of the skin, such as impetigo, erysipelas, and cellulitis [83].

**Figure 3:**
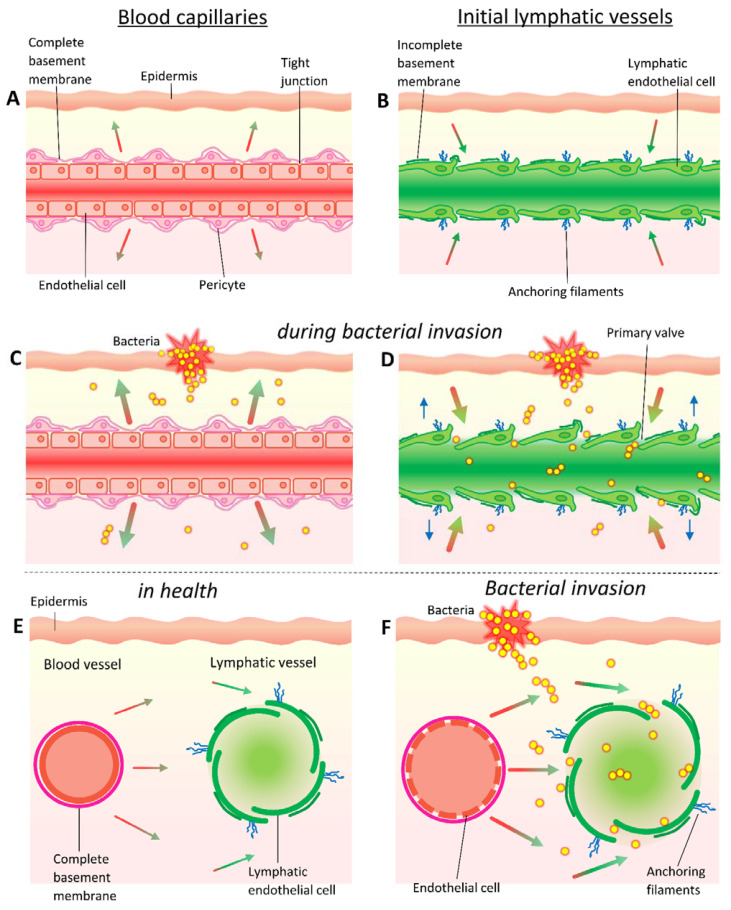
**Fluid exchange and bacterial uptake in the vasculature.** In tissue, fluid leaks from (**A**,**C**) vascular capillaries and venules are characterised by tight junctions between endothelial cells, a complete basement membrane, presence of pericytes, and occasional smooth muscle fibres. By contrast, (**B**,**D**) the initial lymphatics have an incomplete basement membrane, lack smooth muscle, and have buttonlike wide gap junctions between endothelial cells, as well as anchoring filaments that are tethered to surrounding tissue. Though small molecules (≤10 nm diameter) are preferentially absorbed from the interstitial space into the blood capillaries rather than lymphatic capillaries, uptake into the lymphatics rises with increasing molecular size. However, (**C**) bacteria are much too large to passively enter vascular capillaries in most tissues and instead (**D**) pass into the more permeable initial lymphatics. Hydrostatic pressure forces fluid out of capillaries at the arterial end (indicated by red-green gradient filled arrows in (**A**,**C**,**E**,**F**)) which swells the interstitium and causes the tethered lymphatics to open wide gaps between endothelial cells that can accommodate bacteria. These cellular gaps also serve to draw in interstitial fluid (red-green gradient filled arrows in (**B**,**D**–**F**)), including any bacteria present, by increasing intraluminal volume and thus decreasing pressure. During infection and inflammation, (**C**,**F**) plasma leakage resulting from increases in blood vascular permeability which elevates interstitial fluid pressure and rapidly drives (**D**) enhanced lymph flows which increase the speed and efficiency of lymphatic clearance of bacteria from the interstitium.

Initial lymphatics begin as closed-ended stumps (~10–60 μm in diameter) and are formed from a single layer of overlapping oak-leaf shaped endothelial cells which form discontinuous buttonlike junctions and lack a complete basement membrane [93]. The initial lymphatics are tethered to the ECM, and so the swelling of the interstitium resulting from increases in extracellular fluid pressure can open gaps between endothelial cells as large as several micrometres [93,94]. These cellular gaps function as primary valves: as they open, intraluminal volume increases and pressure decreases, drawing in interstitial fluid, particles (such as bacteria), and cells (including dendritic cells); as the lymphatic vessel fills with fluid, the cell junctions are pushed closed again and pressure returns to baseline, preventing fluid from leaking back into the interstitium [93,95,96,97] (Figure 3). Intake of fluid from tissues into the initial lymphatics is also driven by the suction force generated by movement of downstream collecting lymphatics [98].

In addition to the passive uptake of particles, the entry of cells into the initial lymphatics can involve adhesion and junction molecules, chemokine gradients, and intricate multistep integrin-mediated processes [99,100]. The entry of dendritic cells has been demonstrated to be initiated by host hyaluronan-mediated interactions with LYVE-1 in initial lymphatics [97]. Hyaluronan is also produced by several bacterial species, and so hyaluronan-LYVE-1 interactions may augment the lymphatic uptake of these bacteria [21,22]. Indeed, several species of bacteria that possess hyaluronan capsules, including *Pasteurella multocida,* strains of *Bacillus cereus, Streptococcus equi*, and *S. pyogenes* are frequently associated with lymphatic pathologies [22,101,102,103]. Interestingly, *B. cereus* strains which have obtained certain virulence plasmids and acquired the ability to produce hyaluronan capsules become capable of causing fatal *B. anthracis*-like disease, instead of the self-limiting foodborne infections normally associated with *B. cereus* [101].

## 10. Effect of Damage on Vessel Permeability and Fluid Flow

Damage to tissue through trauma, burns, or surgery commonly precedes invasive bacterial infection. As such, it may be considered that antecedent trauma that ruptures blood capillaries which ordinarily exclude large particles could allow bacteria passage inside. However, capillaries are shown to rapidly contract upon injury, and blood typically flows out of injured vessels until they are occluded by clotting [104].

Consistent with these observations, bacteria added to a fresh bleeding wound were not recovered from the circulation of rabbits if downstream lymphatics had been ligated [105]. In contrast, the anchoring of the initial lymphatic to the ECM keeps even damaged lymphatic vessels patent and able to readily uptake particles and bacteria.

Upon tissue injury, mediators are rapidly released which increase blood vascular permeability, primarily by promoting gaps in intracellular junctions of postcapillary venules [106,107]. This results in an increased movement of fluid and plasma proteins, such as complement and antibodies, into the interstitial space [108]. The formation of these endothelial gaps is most common in venules but relatively rare in capillaries [109,110]. Many of the same mediators that modulate blood vessel permeability also target lymphatic vessels [111,112]. Specifically, proinflammatory cytokines including IFN-γ, IL-1β, IL-6, and TNF-α can modulate lymphatic endothelium leakiness in vivo [111,112].

While some endothelial cell gaps in blood vessels are large enough to permit passage of bacteria, as with damaged blood vessels, fluid leaks out of the venule rather than moving inwards [109,110]. This means that these gaps are more relevant to extravasation of particles and the haematogenous seeding of tissue with bacteria, rather than movement of bacteria from tissue to blood. Such a phenomenon may explain the seeding of closed fractures with bacteria [53].

Plasma leakage resulting from increases in blood vascular permeability elevates the interstitial fluid pressure and rapidly drives enhanced lymph flows. In normal conditions, interstitial flow varies in between 0.1 µm/s and 2 µm/s [113], but flow can increase by more than an order of magnitude in inflammation [114]. Fluid shear stress is a modulator of nitric oxide release, leading to increased lymphatic pump function in the collecting lymphatics which elevates flow rate, serving to prevent edema [115]. 

As a consequence of elevated interstitial fluid pressure and lymph flow rate generated by plasma leakage, the speed and efficiency of the lymphatic clearance of bacteria from the interstitium increases. For example, a contusion injury promotes the increased bacterial transit of *S. pyogenes* to draining lymph nodes following subcutaneous infection of mice [116].

## 11. Anatomy of Bacterial Invasion II: Interactions with Interstitial Extracellular Matrix

While selectivity of lymphatic over blood capillary uptake increases with particle size, the rate and efficiency of lymphatic clearance is maximal for particles in the size range between 20 nm and 100 nm, which is much smaller than bacteria (~1–2 µm) [117,118,119]. This threshold corresponds with the ~100 nm diameter of water channels which pervade the interstitial ECM [120,121]. As such, above a size of ~100 nm, the rate and extent of particle clearance from the interstitial space decreases greatly [119]. This physical restriction traps the majority of a bacterial inoculum at the site of inoculation, with only a small proportion of bacteria being observed to disseminate [21,28]. It should be noted that this does not preclude the lymphatic infection route, as multiple studies of bacterial invasive disease have demonstrated tight bottlenecks, with bacteraemia driven by a single organism or small minority of total clones [26,122,123,124,125].

The interstitial ECM itself is an entangled mass of crosslinked collagen fibres, glycosaminoglycans, and glycoproteins which form a porous three-dimensional gel-filled lattice that impedes movement of larger particles and cells [126,127,128]. Although particle size is one of the most important determinants of lymphatic uptake and lymph node retention, particle charge and hydrophobicity, as well as interstitial fluid pressure play important roles [129,130]. These observations likely reflect the degree of interstitial hindrance a particle experiences prior to lymphatic uptake.

The ECM possesses a negative charge and hydrophilic properties, conferred by the presence of the glycosaminoglycan hyaluronan [131,132,133]. Therefore, particles that also carry a net negative or neutral charge and possess hydrophilic properties exhibit an electrostatic repulsion to the ECM. This repulsion increases migration through the interstitium and improves lymphatic uptake, whereas positively charged and hydrophobic particles exhibit the opposite characteristics [120,134,135].

These physiochemical properties also play an important role in the invasiveness of bacteria and likely influence the efficiency of bacterial lymphatic dissemination (as discussed in the next Section 12. Properties of Bacteria in Invasion). Many bacteria release collagenases, hyaluronidases, and other enzymes that can degrade the ECM and facilitate bacterial movement and spread [136]. Additionally, the inflammatory host response to bacteria directs the remodelling of the ECM, which is mediated through digestion of ECM components by proteases secreted by recruited neutrophils, as well as tissue-resident cells [137]. Such degradation of the ECM likely decreases hindrance for infiltrating immune cells, but may also increase the dissemination of bacteria present, as is observed in increased metastasis of tumours in cancer [137,138].

## 12. How Bacterial Properties Influence Invasion

Many bacterial species produce exopolysaccharides (including capsules) which are typically hydrophilic and negatively charged. These exopolysaccharides can increase electrostatic repulsion to the extracellular matrix [134], mask underlying adhesion molecules on the bacterial surface [139,140], inhibit phagocytic clearance [141], and ultimately enhance uptake into lymphatic vessels [21,23,129,130]. Certain exopolysaccharides can also play additional roles in invasion, such as the hyaluronan capsule of *S. pyogenes*, which can open the cell junctions of keratinocytes in the skin through CD44-mediated cell signaling [86] and may also augment entry into the initial lymphatics through interaction with LYVE-1 [21,22].

Hydrophobicity is one of the most important factors in microbial attachment to a surface and bacterial adhesion to tissues is largely mediated by outer membrane proteins through hydrophobic and other interactions [50,142,143,144]. This includes adhesion to epithelial and endothelial cell surfaces, as well as ECM. Though adherence can be important for virulence, encapsulated or mucoid strains are typically more virulent despite being inherently less tissue adhesive [21,23,47]. For instance, non-encapsulated strains of *Haemophilus influenzae* are much more commonly identified in nasopharyngeal carriage than encapsulated strains, but only the latter are strongly linked with bacteraemia [47,145].

The association of encapsulated strains with hypervirulence and bacteraemia is commonly attributed to improved resistance to phagocytosis and complement attack, as well as evasion of adaptive immune elements [47]. While these properties are undoubtably important, increased lymphatic uptake is also likely to play an important role in the hypervirulence of many encapsulated strains. As encapsulation reduces non-specific bacterial interactions with host cells and surfaces, encapsulated strains are less likely to adhere to the ECM or cells in the periphery and are thus more likely to enter lymphatic vessels and reach the systemic circulation. This suggests that passive uptake into the lymphatics is important to instigate bacteraemia. Infection experiments using encapsulated and non-encapsulated bacteria in phagocyte-depleted hosts have the potential to uncouple the relative contributions of increased lymphatic uptake and phagocytosis resistance to hypervirulence.

Neither encapsulation nor hydrophobicity are necessarily dichotomous: within a population of bacteria, individual bacterial cells with differing levels of protein, exopolysaccharide, and resultant hydrophobicity are present. Expression of cell surface characteristics determining adhesion, including exopolysaccharide, are influenced by growth conditions in many bacteria [146,147]. The interaction of bacteria with tissues can also alter expression profiles: the introduction of *S. pyogenes* into the pharynx or deep tissues of mice results in a rapid induction of capsule expression [147]. Therefore, a bacterial inoculum may be able to initially adhere to host tissue and colonize a host, but subsequently some of these bacteria could alter their physiochemical properties to those better suited to dissemination. Alternatively, adherent subpopulations of bacteria could invade and damage epithelial cell layers, with other less adherent subpopulations better able to pass through the disrupted barrier and disseminate through the lymphatics.

## 13. Anatomy of Bacterial Invasion III: Path of Lymph Return to the Blood

Once interstitial fluid has left the tissue and entered the initial lymphatics it is characterised as lymph and flows unidirectionally away from the periphery into progressively larger lymphatic vessels, termed collecting lymphatics.

Collecting lymphatic vessels have their endothelial cells arranged in a continuous zipper junction formation and are surrounded by a complete basement membrane which greatly decreases permeability compared to initial lymphatics and reduces any intake or egress of lymph and particles [148]. As such, collecting lymphatics function primarily as conduits, as opposed to contributing to lymph formation [93], although, inflammation can enhance permeability and permit lymph uptake as well as transmigration of immune cells [149]. There is not a distinct border between initial and collecting lymphatics and the term pre-collecting lymphatics is commonly used to refer to intermediate sections which share features of both vessels and possess intermediate permeability; this means that bacteria in deeper tissue may sometimes be able to enter lymph via pre-collecting lymphatics, particularly during inflammation [150]. Collecting lymphatics actively pump, which combined with intermittent squeezing from skeletal muscle, breathing, and other sources, propels lymph forward through regularly spaced valves that prevent backflow [88]. These efferent lymphatics eventually converge to create lymphatic trunks, which then drain into one of the two large lymphatic ducts. The thoracic lymphatic duct is the largest lymphatic vessel in the body and receives all draining lymph, bar that originating in the right thorax, arm, head and neck, which instead flows into the right lymphatic duct. These lymphatic ducts return lymph into the bloodstream via the left and right subclavian veins, respectively. Finally, all lymph fluid is returned into the heart via the vena cava.

Bacteria can follow the entire path of lymph to transit rapidly from peripheral tissue to the blood circulation [21]. However, all lymph first passes through at least one of the roughly 600 lymph nodes in the human body [151]. Lymph nodes are strategically organised along collecting lymphatics from key sites of lymph drainage. They are often arranged in series and so lymph from certain tissues may have to pass through multiple lymph nodes before reaching the blood, which improves filtration efficiency [88,152].

Despite the sometimes sluggish pace of interstitial fluid flow in healthy tissue at rest, injected antigens and bacteria can reach lymph nodes in minutes [21,23,153,154]. Injection volume elevates interstitial tissue pressure and lymph flow in experimental infection models. Even though in natural infection, inoculation of bacteria occurs without an appreciable volume of liquid, onset of inflammation and formation of oedema augment lymph flow [155,156]. Such rises heighten the lymphatic uptake of bacteria, as observed with increased transit of intramuscularly inoculated *S. pyogenes* to local draining lymph nodes following mild contusion injury [116].

## 14. Anatomy of Bacterial Invasion IV: Path of Lymph through the Lymph Node

Lymph enters the lymph node through one of several afferent collecting lymphatic vessels and moves around the perimeter of the lymph node in the subcapsular sinus (SCS) which is lined with macrophages that express high amounts of CD169 (CD169^High^) and span the SCS endothelium in a transcellular arrangement [19,157].

Small particles (<5 nm) and molecules (<70 kDa) may pass out of the subcapsular sinuses into the ECM-like conduit system of the lymph node (permitting direct delivery of antigens and cytokines to deeper B and T cell zones). However, above this size, the passage of particles through the floor of subcapsular sinus-lining endothelium into parenchymal conduits is physically restricted by a diaphragm comprised of the plasmalemma vesicle–associated protein (PLVAP) that covers transendothelial channels [158].

Therefore, particles not binding to SCS macrophages are carried onward with lymph flow into the meandering and often interconnected LYVE-1^+^ medullary sinuses [159]. As with injected dyes and particles, bacteria accumulate in this region [21,35], however, those that do not are carried with lymph into the singular efferent lymphatic, which is an onward pathway to the blood. For entry into the lymphatic system to not represent a dead-end in invasion, bacteria must be able either to pass through filtering lymph nodes with sufficient efficiency, replicate well within them, or simply persist and resist clearance.

## 15. Immune Defence in Lymph Nodes

Although lymph nodes are best known for their role in sampling antigens from tissues and coordinating durable adaptive immune responses [160,161], their contribution to innate defence against bacteria through the actions of resident and recruited phagocytes was first identified in the late 19th century [6,162,163]. As such, bacteria surviving within the lymph node must possess suitable virulence factors to resist phagocytic killing through either avoidance of phagocytosis or survival upon phagocytosis [164].

In recent years, there has been a finer elucidation of the antimicrobial mechanisms of the lymph node using updated molecular methods. Coordinated responses by distinct niches of lymphatic endothelial cells, SCS and medullary sinus (MS) macrophages, monocytes, neutrophils, natural killer cells, and other innate lymphocytes, controlled by cytokines and other mediators are reported to attempt to constrain infection within the lymph node [18,19,35,165,166,167,168,169]. However, many of these studies have used viral infection models. Data on responses to strains of virulent live bacteria, which are likely to differ substantially from viral responses, are much more incomplete.

Though depletion of lymph node macrophages using clodronate liposomes increased the bacterial load of *P. aeruginosa* in the lymph nodes and blood of mice [35], abrogation of these macrophages had no effect in a mouse model of *S. pyogenes* infection [23]. Interestingly, in a model of *S. aureus* infection, in which bacteria were not ordinarily recovered from the systemic organs, inhibition of neutrophil recruitment to lymph nodes greatly increased the systemic bacterial loads compared to a modest effect obtained by the depletion of macrophages [36].

## 16. Lymph Nodes as Bacterial Filters

In addition to cellular defences, the meandering sinuses of the lymph node medulla act as baffles, slowing lymph flow and resulting in the settling of bacteria [6,170], evidenced by the accumulation of cell-free bacteria independent of phagocytes in lymph node medullary sinuses [21,35]. Similar bacterial filtration efficiency has been reported irrespective of observed phagocytosis [6]. Therefore, mechanical factors, such as lymph flow rate and obstruction of lymphatic vessels and sinuses could explain the slowing in the passage of bacteria. Combined, these mechanisms appear to restrict—but typically not abrogate—the movement of bacteria through lymph nodes.

Quantitative assessment of the efficiency of lymph node filtration of bacteria has rarely been performed, with reported results ranging from ~99% for some strains of *S. pyogenes*, *S. aureus*, and *B. anthracis*, to ~80% for *Bacillus subtilis* [5,6,171]. Lymph flow can impact filtration efficiency, with increased flow upping the proportion of bacteria escaping the lymph node [171]. It has also been suggested that the highly concentrated bacterial preparations used in the above experiments may result in “clogging” of sinuses and artificially increase the efficiency of lymph node filtration, such that lower bacterial numbers, that might be expected in natural infections, would be less efficiently held within the lymph node [171].

In shorter time course experiments, bacterial passage through the lymph node has been reported to be greatest in the initial minutes or hours following injection [163,171]. These factors indicate that an early rush of bacteria may be able to disseminate through the lymph node with great efficiency before cellular defences respond.

## 17. Role and Recruitment of Neutrophils in Lymph Nodes

Increased filtration efficiency over time coincides with the inflammatory response in the lymph node [163]. As with bacterial infections at other sites in the body, neutrophils are first responders in the lymph node and seem to be key players in the bactericidal response.

There is a striking recruitment of neutrophils observed following the arrival of wide array of bacteria to a draining lymph node, including *S. pyogenes* [21], *S. aureus* [67,172], *P. aeruginosa* [35], *Y. pestis* [15], and *M. bovis* [17]. Neutrophils enter draining lymph nodes within 15 minutes of the arrival of bacteria and their numbers peak in the hours that follow in line with the development of bacterial infection [21,36,67,171]. These recruited neutrophils appear to home in on populations of bacteria present in the SCS and, particularly, medullary sinuses, forming swarms [21,36,172,173]. Lymphatic endothelial cells lining the subcapsular floor and the medullary sinuses express multiple neutrophil chemoattractants, [167]. The targeting of bacterial dense regions within sinuses could be mediated by the lymphatic endothelial cells lining the lymph node sinuses which express neutrophil chemoattractants, including C-type lectin CD209 [167]. Distinct expression patterns of different types of lymphatic endothelial cells could also be important in bacterial interactions within lymph node niches [168].

Following intradermal injection of pneumococci, roughly 50% of cells in the afferent lymphatic of the local draining lymph node are neutrophils [163]. Neutrophils have also been observed in these vessels after infection with other bacteria, including *S. aureus* and *M. bovis* [17,174]. However, lymphatic vessels draining an infection site contribute only a fraction of the total neutrophils recruited to the lymph node, which principally enter through blood vessels [163]. This recruitment occurs through high endothelial venules via L- and P-selectin-dependent mechanisms and is expediated by a small population of distinct neutrophils that recirculate through the lymph node parenchyma [36,172].

Several, potentially redundant, mechanisms appear to be involved in neutrophil recruitment to lymph nodes during infection. Complement component C5a has been identified as a prominent neutrophil chemoattractant following *S. aureus* infection [36]. In *S. pyogenes* infection, chemokine CXCL8 (CXCL1/2 in mice) contributes to neutrophil recruitment [21,175]. Neutrophil recruitment to the lymph node following *P. aeruginosa* infection is reduced in IL-1R knockout mice and has also been suggested to be enhanced by caspase-1-dependent IL-1β production by both SCS and MS macrophages following inflammasome activation [35].

## 18. Subcapsular Sinus Macrophages in the Lymph Node

As lymph flows around the subcapsular sinus, it flows past CD169^High^ SCS macrophages. SCS macrophages are poorly endocytic and have weak lysosomal activity; instead, these cells appear to be most important in antigen presentation [176,177,178]. SCS macrophages can capture immune complexes, antigens and some viruses transiting in lymph and retain these moieties on their surface, before translocating them below the SCS floor for display to adjacent follicular B cells [18,176,179].

Despite weak phagocytic capabilities, SCS macrophages have been implicated in pathogen control. The binding of some viruses by SCS macrophages has been reported to limit invasion by restricting viral transit through the lymph node [18,35,180]. This so called “cellular flypaper” mechanism is also frequently believed to limit dissemination in bacterial infection despite a lack of direct supporting evidence. Though, parallels do exist in the spleen: splenic marginal zone (MZ) macrophages, which also express CD169, have been reported to capture *S. pneumoniae* which facilitates subsequent clearance by neutrophils [60]. Equally, *S. pneumoniae* has recently been shown to replicate inside of MZ macrophages in vivo, which functions as an intracellular haven to drive later bacteraemia [64].

Work by Kastenmüller et al. is often cited as a demonstration of the relevance of SCS macrophage “cellular flypaper” in control of bacterial infection in the lymph node, but close appraisal of the data presented in this paper shows it to be largely inconsistent with that interpretation [35]. Firstly, significant bacterial systemic dissemination was still observed prior to macrophage depletion, demonstrating that the presence of macrophages does not prevent passage of the studied strain of *Pseudomonas aeruginosa* through the lymph nodes. Secondly, both before and after depletion of macrophages, the unnamed strain of *P. aeruginosa* assessed appeared to accumulate predominantly in the medullary sinuses rather than the SCS, demonstrating that SCS macrophages failed to prevent bacterial passage from the SCS into the medulla. Finally, clodronate depletes phagocytic MS macrophages as well as SCS macrophages, meaning it is not possible to distinguish between the contribution of these two subsets in regard to the resultant elevated bacterial load in the local draining lymph nodes following clodronate depletion of macrophages [21].

SCS macrophages do possess some receptors that could in theory capture some types of bacteria from flowing lymph; the aforementioned CD169 can bind sialylated bacteria, including meningococcus, group B streptococcus, and *Campylobacter jejuni*. However, CD169-mediated bacterial capture has not been demonstrated within lymph nodes [181,182,183]. SCS macrophages also express complement receptor 3 on their surface, which allows the binding of opsonised particulates with enhanced efficiency [18,184], although internalisation and degradation of opsonised antigens remains low [185]. Lastly, mannose-binding lectin, which can bind to multiple bacterial species, has been demonstrated to mediate the binding of influenza virus to SCS macrophages, also likely via complement receptors [186]. Again, this has not been demonstrated to mediate capture of bacteria on SCS macrophages, illustrating a clear knowledge gap regarding specific bacterial–host cell interactions in the lymph node. Though, as virulent bacteria typically have mechanisms to restrict opsonisation by complement [187,188,189], particularly in the absence of specific antibodies, it might be anticipated that SCS macrophages would not capture virulent bacteria efficiently.

While Kastenmüller et al. do not provide good evidence in support of cellular flypaper capture of bacteria by SCS macrophages in the lymph node, the manuscript does demonstrate that lymph node macrophages contribute antibacterial activity against *P. aeruginosa*. Prestored IL-18, present in both SCS and MS macrophages and released upon inflammasome activation is required for the production of IFNγ by closely associated innate lymphocytes, which exerts a slight antibacterial effect [35]. However, IFNγ-producing innate lymphocytes are present in greater numbers in the medulla than near the subcapsular sinus, indicating that MS macrophages are more important in orchestrating this modality of immunity.

## 19. Medullary Sinus Macrophages in the Lymph Node

After flowing around the subcapsular sinus, large particles, including bacteria, reach and commonly accumulate within the medullary sinuses [21,35], which contain many MS macrophages. MS macrophages share CD169 expression with SCS macrophages (albeit at lower levels) but are distinguished by their F4/80 expression and differ greatly in function. In contrast to SCS macrophages, MS macrophages are highly phagocytic, possessing larger lysosomes, and expressing greater levels of degradative enzymes than SCS macrophages [157,176].

MS macrophages, and some sinus-lining lymphatic endothelial cells, can also express several surface receptors that recognise some polysaccharide bacterial antigens, including SIGN-R1, LYVE-1 and MARCO [20,190,191]. A few of these receptors have been demonstrated to mediate the capture or internalisation of bacteria in the spleen or cell culture. SIGN-R1 mediates the binding of the capsular polysaccharide of heat-killed *Streptococcus pneumoniae* by splenic marginal zone macrophages and contributes to protection against lethal pneumococcal infection in mice [192,193]. Although, once again, bacterial binding has not been characterised in the lymph node. LYVE-1, which is expressed by some MS macrophages, binds encapsulated *S. pyogenes* in transfected cell lines and so could play a role in the capture of such hyaluronan-expressing bacteria in vivo [21,22]. The scavenger receptor MARCO is upregulated by microbial stimuli in a TLR-dependent manner and involved in the capture of heat-killed *Escherichia coli* and *Staphylococcus aureus* by macrophages in the marginal zone of the spleen [194,195]. Interestingly, blocking MARCO only inhibits the elimination of heat-killed bacteria from the blood and not live *E. coli* or and *S. aureus* [194]. As mentioned previously, studies investigating bacterial capture by macrophages often only assessed killed bacteria or bacterial antigens. The discrepancy between the MARCO-mediated capture of killed and live bacteria further highlights the need to further investigate capture of live bacteria within the lymph node.

## 20. Bacterial Survival in the Lymph Node

Interference with the recruitment and actions of neutrophils and macrophages are important virulence strategies for strains of pathogenic bacteria. These mechanisms are important for survival within an inflamed lymph node. For example, *S. pyogenes* with mutations in key streptococcal virulence regulator CovR/S obtains a hypervirulent phenotype with increases in expression of multiple virulence factors, including hyaluronan capsule, *S. pyogenes* chemokine-cleaving protease (SpyCEP), as well as C5a peptidase (ScpA) [21]. These factors reduce CXCL1/2- and C5a-mediated neutrophil recruitment, in addition to inhibiting phagocytosis by neutrophils and macrophages [21].

**Figure 4:**
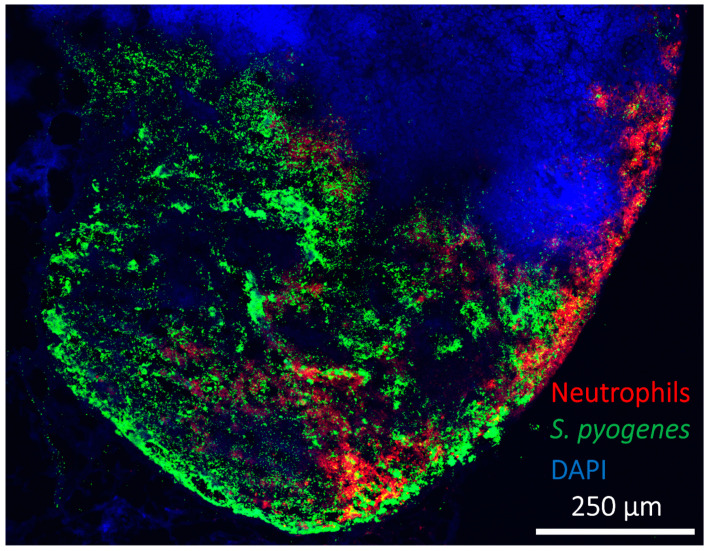
Expansion of virulent bacteria in a murine draining lymph node. Despite strong recruitment of phagocytic neutrophils (red), bacteria (*S. pyogenes*: green) can rapidly expand from a much smaller initial population in around 24 hours post infection at a distant site (intramuscular) [21]. DAPI staining of nuclei (blue) demonstrates extensive necrotic regions, focussed in the medulla and regions of the subcapsular sinus, where bacteria are present and prominent lysis of host cells has occurred. Adapted from Siggins et al., 2020 [21].

When injected intramuscularly into the leg of a mouse, hypervirulent *S. pyogenes* demonstrated early movement through sequential draining lymph nodes. In addition, the hypervirulent bacteria was able to replicate in lymph nodes unchecked, resulting in the formation of metastatic foci of spreading necrosis which appeared to compromise the lymph node structure within 24 hours of infection and coincided with severe and lethal bacteraemia [21] (Figure 4). Others have also reported *S. pyogenes* existing predominantly extracellularly in lymph nodes [196]. Similar foci are reported in lymph nodes after infection with other bacteria, including *B. anthracis* and *Y. pestis,* which can flood the bloodstream via the lymphatics after hours to days of the initiation of infection [24,40,197]. For a wide range of bacterial species, the numbers of viable bacteria recovered from lymph nodes correlate with virulence and the ability to subvert phagocytes, with the production of an exopolysaccharide is often important [21,23,198].

Resistance to phagocytosis and phagocytic killing is certainly important for bacterial fate within lymph nodes. As in other areas of the body, including the blood, the survival and propagation of invading bacteria is highly dependent on the elaboration of specific virulence factors and overall virulence. Relatively avirulent bacteria can be killed by neutrophils within minutes, the killing of *S. pyogenes* by either human or mouse neutrophils was reported to be complete within 30 minutes [199,200,201], though exact timing is likely to vary based on bacterial and experimental conditions. Accordingly, certain strains of bacteria are promptly phagocytosed and digested within hours in the lymph node, whereas other bacteria can persist or even accumulate significantly over 24 hours [21,24,163].

As well as differences in lymphatic survival between bacterial species, there is considerable variation in survival between strains of the same species, and even identical strains grown in different conditions [6,21,23]. We found that mutants of the same strain of a clinical *emm*1 *S. pyogenes* isolate, that differed only in levels of hyaluronan capsule production, had significantly different levels and kinetics of lymphatic persistence. In these experiments, an acapsular *emm1* strain was largely cleared from draining lymph nodes within 24 hours, which contrasted sharply with an otherwise identical hyper-encapsulated strain that persisted at peak levels for at least 24 hours [21].

## 21. Impact of Bacteria in Lymph Nodes on Immunity

By design, many animal infection models consistently result in systemic infections after the infection of healthy animals with virulent bacteria. However, severe systemic bacterial infections are rare in humans; instead, repeated minor bacterial infections are much more common. Lymph nodes are important to develop robust adaptive immunity against pathogens, particularly through the formation of germinal centres, which generate long-lived plasma cells that produce antibodies to maintain long-lasting sterilizing immunity [160]. As such, the main influence of bacterial lymphatic metastasis could be the disruption of the cognate host immune response through bacterial persistence in draining lymph nodes. In this way, bacterial lymphatic metastasis may contribute to recurrent childhood streptococcal infections by slowing the natural development of protective immunity [202].

*S. aureus* has been reported by several investigators to interfere with immune responses when present in lymph nodes, likely due to dysregulation prompted by concurrent inflammation [66,67,174]. *S. pyogenes* has been shown to reduce the size of germinal centres in tonsils, and *Salmonella* bacterial burden delays germinal centre formation in the spleen [68]. Superantigens play a role in the dysregulation of immune function in lymphoid tissue seen upon infection with *S. pyogenes* [203,204]. Superantigens are secreted toxins, also produced by *S. aureus*, which activate T cells in an uncontrolled manner and can dysregulate tonsil immune function, partly by directing T follicular helper cell-mediated killing of B cells in lymphoid tissue [203,204]. As mentioned, we have measured superantigens in lymph nodes after staphylococcal soft tissue infection [69]. Notably, superantigens are of a sufficiently small size to pass through lymph node sinuses, where we observed bacteria to accumulate, to deeper cortical areas, enabling direct interaction with lymphocytes participating in active immune responses.

Heavily encapsulated *S. pyogenes* isolates are associated with the autoimmune disease, rheumatic fever [205], raising the possibility that the strong lymphatic-retention of encapsulated isolates, mediated in part by LYVE-1 adhesion, provides a persistent stimulus that leads to development of autoimmunity. Additionally, interaction between streptococci and lymphocytes within the efferent lymphatics, possibly mediated through bacterial adhesion to CD44, could be anticipated to have consequences for immunity [21]. Alternatively, extracellular bacterial dissemination to draining lymph nodes may actually be an important part of developing strong responses to bacterial pathogens. Bacterial metastasis would deliver unprocessed antigens on live bacteria directly to the lymph node, with far-reaching implications for the modelling of innate and adaptive immunity.

A higher antigen concentration in lymph nodes can result in stronger immune responses, thus transit of bacteria in lymph flow to lymph nodes could be important for the generation of robust immunity during natural infection. Additionally, the enhanced lymphatic-homing and retention mediated by hyaluronan could be harnessed to improve antigen delivery to lymph nodes. Accordingly, we are currently investigating LYVE-1-expressing avirulent bacterial vectors as an approach to generate enhanced vaccine responses against pathogenic bacteria.

## 22. Conclusions and Future Perspectives

Upon comprehensive review of the literature, there can be little doubt that lymphatic bacterial metastasis is a real phenomenon that often predominates over vascular invasion and drives systemic infection in a large array of experimental animal models. Equally, even less invasive bacteria appear able to frequently reach draining lymph nodes in sufficient numbers to suggest that the interaction is of consequence. Although not universally observed, a diverse breadth of bacteria, administered by a range of injection routes, appear to exhibit lymphatic-dominated dissemination patterns. This should prompt the reconsideration of currently understood paradigms of bacterial dissemination, pathogenesis, and immunity.

Convincing demonstration of lymphatic dissemination has relied on animal models due to the invasive nature of the required experimental techniques. Clearly there are many physiological and immunological differences between animals, such as mice, and man, including differences in the bactericidal activity of blood and host responses to the presence of bacteria in blood [206,207,208,209]. However, despite a lack of conclusive direct proof that bacterial metastasis has relevance in human infection, its compatibility with the clinical picture of fatal invasive infections that lack an identifiable initial site of infection strongly encourages further experimental investigation. Such research has the potential to generate a groundbreaking impact in the fields of infection and immunology.

## Figures and Tables

**Table 1:** Studies demonstrating the recovery of bacteria from the local draining lymph nodes following infection.

Bacteria	Infection Route	Model	Mechanisms of Transit to Local Draining Lymph Nodes	Recovery of Bacteria from Other Lymphatic Sites	References
*Bacillus anthracis* (spores)	Aerosol, ID, IP	Rhesus macaque	Unknown	Efferent lymph	Lincoln, R.E. 1965; [24]
*Escherichia coli*	OG	Mouse	Unknown	None investigated	Balmer, M. 2014; [29]
*Francisella tularensis*	IN	Mouse	Extracellular and intracellular within dendritic cells	None investigated	Bar-Haim, E. 2008; [30]
*Klebsiella pneumoniae*	IM	Mouse	Unknown	Distant draining lymph nodes	Siggins, M.K. 2019; [23]
*Listeria monocytogenes*	IG, FB Footpad	Mouse	Unknown, influence of bacterial E-cadherin ligand Unknown	None investigated	Bou Ghanem, E. 2012; [31] St. John, A.L. 2009; [32]
*Mycobacterium tuberculosis*	Aerosol	Mouse Cynomolgus and Rhesus macaque	Unknown	None investigated	Chackerian, A.A. 2002; [33] Ganchua, S.K. 2018; [34]
*Pseudomonas aeruginosa*	IM Footpad	Mouse	Unknown	Distant draining lymph nodes None investigated	Siggins, M.K. 2019; [23] Kastenmüller, W. 2012; [35]
*Salmonella* Abortusovis	SC (oral)	Sheep	Free extracellular or associated with monocytes and granulocytes	Afferent lymph	Bonneau, M. 2006; [16]
*Salmonella* Typhimurium	OG Footpad	Sheep Mouse	Free extracellular or associated with dendritic cells Unknown	Afferent lymph None investigated	Bravo-Blas, A. 2019; [25] St. John, A.L. 2009; [32]
*Staphylococcus aureus*	IM	Mouse	Unknown	None investigated	Bogoslowski, A. 2018; [36]
*Streptococcus agalactiae*	IM	Mouse	Unknown, influence of capsule	Distant draining lymph nodes	Siggins, M.K. 2021; [unpublished]
*Streptococcus pyogenes*	IM	Mouse	Free extracellular, influence of HA capsule and LYVE-1 interaction	Distant draining lymph nodes and efferent lymph None investigated	Siggins, M.K. 2020; [21] Lynskey, N.N. 2015; [39]
*Yersinia pestis*	ID Footpad	Mouse	Unknown but not dependent on neutrophils or dendritic cells Free extracellular independent of phagocytes Free extracellular and in mononuclear phagocytes	None investigated Afferent lymphatic Distant draining lymph node and efferent lymphatic	Shannon, J.G. 2013; [28] Gonzalez, R.J. 2015; [26] St. John, A.L. 2014; [15]

FB: foodborne; ID: intradermal; IG: intragastric; IM: intramuscular; IN: intranasal; IP: intraperitoneal; OG: oral gavage; SC: subcutaneous.

**Table 2:** Studies demonstrating recovery of bacteria from the lymphatic system beyond local draining lymph nodes.

Bacteria	Infection Route	Model	Sites of Recovery beyond Local Draining Lymph Nodes	Predominant Mechanisms of Lymphatic Transit Reported	References
*Bacillus anthracis* (spores)	Aerosol, IP, and SC Aerosol and SC	Rhesus macaque Rabbit	Efferent lymph	Unknown	Lincoln, R.E. 1965; [24] Hughes, R. 1956; [40]
*Klebsiella pneumoniae*	IM	Mouse	Distant draining lymph nodes	Unknown	Siggins, M.K. 2019; [23]
*Pseudomonas aeruginosa*	IM	Mouse	Distant draining lymph nodes	Unknown	Siggins, M.K. 2019; [23]
*Salmonella* Dublin	Oral	Cow	Efferent lymph	Free extracellular	Pullinger, G.D. 2007, [38]
*Streptococcus pyogenes*	IM	Mouse	Distant draining lymph nodes and efferent lymphatics	Free extracellular and extracellular association with leukocytes, contribution of HA capsule and interaction with LYVE-1	Siggins, M.K. 2020; [21]
*Streptococcus pneumoniae*	IN and IT	Rabbit	Efferent lymph	Free extracellular	Schulz, R.Z. 1938; [37]
*Yersinia pestis*	Footpad	Mouse	Distant draining lymph nodes and efferent lymphatic	In mononuclear phagocytes	St. John, A.L. 2014; [15]

IM: intramuscular; IN: intranasal; IP: intraperitoneal; IT: intratracheal; SC: subcutaneous.

**Table 3:** Infections with known lymphatic involvement or potential as a lymphatic portal.

Term	Site	Description
Bacteraemia	Blood	Presence of viable bacteria in the circulating blood
Cellulitis	Subcutaneous tissue	Infection of the lower dermis and subcutaneous tissue causing spreading inflammation
Cryptic infection	-	Bloodstream or deeper tissue infections that lack a known peripheral focus
Erysipelas	Epidermis	Infection of the superficial epidermis
Fasciitis (necrotizing)	Fascia	Infection of superficial or deep fascia in association with tissue destruction.
Impetigo	Keratin layer of epidermis	Infection of superficial keratin layer of skin
Lymphadenitis/lymphadenopathy	Lymph nodes	Inflammation/enlargement of lymph nodes due to presence of infection within vessels or tissue drained by the lymph node, or the node itself
Lymphangitis	Lymphatic vessels	Inflammation of lymphatic vessels resulting from infection of the vessels themselves, or nearby tissue
Myositis/myonecrosis	Muscle	Infection of muscle leading to inflammation/necrosis
Puerperal sepsis	Uterus	Infection of the genital tract, particularly the uterus, occurring soon after labour

## Data Availability

Not applicable.

## References

[1] Fardini Y., Wang X., Temoin S., Nithianantham S., Lee D., Shoham M., Han Y.W. (2011). Fusobacterium nucleatum adhesin FadA binds vascular endothelial cadherin and alters endothelial integrity. Mol. Microbiol..

[2] Lemichez E., Lecuit M., Nassif X., Bourdoulous S. (2010). Breaking the wall: Targeting of the endothelium by pathogenic bacteria. Nat. Rev. Microbiol..

[3] Spadoni I., Zagato E., Bertocchi A., Paolinelli R., Hot E., Di Sabatino A., Caprioli F., Bottiglieri L., Oldani A., Viale G. (2015). A gut-vascular barrier controls the systemic dissemination of bacteria. Science.

[4] Rangel S.M., Diaz M.H., Knoten C.A., Zhang A., Hauser A.R. (2015). The Role of ExoS in Dissemination of Pseudomonas aeruginosa during Pneumonia. PLoS Pathog..

[5] Clement C.C., Wang W., Dzieciatkowska M., Cortese M., Hansen K.C., Becerra A., Thangaswamy S., Nizamutdinova I., Moon J.Y., Stern L.J. (2018). Quantitative Profiling of the Lymph Node Clearance Capacity. Sci. Rep..

[6] Drinker C.K., Field M.E., Ward H.K. (1934). The Filtering Capacity of Lymph Nodes. J. Exp. Med..

[7] DeLong T.G., Simmons R.L. (1982). Role of lymphatic vessels in bacterial clearance from early soft-tissue infection. Arch. Surg..

[8] Macpherson A.J., Uhr T. (2004). Induction of protective IgA by intestinal dendritic cells carrying commensal bacteria. Science.

[9] Ribet D., Cossart P. (2015). How bacterial pathogens colonize their hosts and invade deeper tissues. Microbes Infect..

[10] Lubkin A., Torres V.J. (2017). Bacteria and endothelial cells: A toxic relationship. Curr. Opin. Microbiol..

[11] Casadevall A., Fang F.C. (2020). The intracellular pathogen concept. Mol. Microbiol..

[12] Banerji S., Ni J., Wang S.X., Clasper S., Su J., Tammi R., Jones M., Jackson D.G. (1999). LYVE-1, a new homologue of the CD44 glycoprotein, is a lymph-specific receptor for hyaluronan. J. Cell Biol..

[13] Oliver G., Kipnis J., Randolph G.J., Harvey N.L. (2020). The Lymphatic Vasculature in the 21(st) Century: Novel Functional Roles in Homeostasis and Disease. Cell.

[14] Oliver G., Detmar M. (2002). The rediscovery of the lymphatic system: Old and new insights into the development and biological function of the lymphatic vasculature. Genes Dev..

[15] St John A.L., Ang W.X.G., Huang M.N., Kunder C.A., Chan E.W., Gunn M.D., Abraham S.N. (2014). S1P-Dependent trafficking of intracellular yersinia pestis through lymph nodes establishes Buboes and systemic infection. Immunity.

[16] Bonneau M., Epardaud M., Payot F., Niborski V., Thoulouze M.I., Bernex F., Charley B., Riffault S., Guilloteau L.A., Schwartz-Cornil I. (2006). Migratory monocytes and granulocytes are major lymphatic carriers of Salmonella from tissue to draining lymph node. J. Leukoc. Biol..

[17] Abadie V., Badell E., Douillard P., Ensergueix D., Leenen P.J., Tanguy M., Fiette L., Saeland S., Gicquel B., Winter N. (2005). Neutrophils rapidly migrate via lymphatics after Mycobacterium bovis BCG intradermal vaccination and shuttle live bacilli to the draining lymph nodes. Blood.

[18] Junt T., Moseman E.A., Iannacone M., Massberg S., Lang P.A., Boes M., Fink K., Henrickson S.E., Shayakhmetov D.M., Di Paolo N.C. (2007). Subcapsular sinus macrophages in lymph nodes clear lymph-borne viruses and present them to antiviral B cells. Nature.

[19] Iannacone M., Moseman E.A., Tonti E., Bosurgi L., Junt T., Henrickson S.E., Whelan S.P., Guidotti L.G., von Andrian U.H. (2010). Subcapsular sinus macrophages prevent CNS invasion on peripheral infection with a neurotropic virus. Nature.

[20] Elomaa O., Kangas M., Sahlberg C., Tuukkanen J., Sormunen R., Liakka A., Thesleff I., Kraal G., Tryggvason K. (1995). Cloning of a novel bacteria-binding receptor structurally related to scavenger receptors and expressed in a subset of macrophages. Cell.

[21] Siggins M.K., Lynskey N.N., Lamb L.E., Johnson L.A., Huse K.K., Pearson M., Banerji S., Turner C.E., Woollard K., Jackson D.G. (2020). Extracellular bacterial lymphatic metastasis drives *Streptococcus pyogenes* systemic infection. Nat. Commun..

[22] Lynskey N.N., Banerji S., Johnson L.A., Holder K.A., Reglinski M., Wing P.A., Rigby D., Jackson D.G., Sriskandan S. (2015). Rapid Lymphatic Dissemination of Encapsulated Group A Streptococci via Lymphatic Vessel Endothelial Receptor-1 Interaction. PLoS Pathog..

[23] Siggins M.K., Lynskey N.N., Lamb L.E., Johnson L.A., Huse K.K., Pearson M., Banerji S., Turner C.E., Woollard K., Jackson D.G. Lymphatic Metastasis of Virulent Extracellular Bacteria Drives Systemic Infection. SSRN Cell Sneak Peek 2019. https://papers.ssrn.com/sol3/papers.cfm?abstract_id=3380255.

[24] Lincoln R.E., Hodges D.R., Klein F., Mahlandt B.G., Jones W.I., Haines B.W., Rhian M.A., Walker J.S. (1965). Role of the lymphatics in the pathogenesis of anthrax. J. Infect. Dis..

[25] Bravo-Blas A., Utriainen L., Clay S.L., Kastele V., Cerovic V., Cunningham A.F., Henderson I.R., Wall D.M., Milling S.W.F. (2019). Salmonella enterica Serovar Typhimurium Travels to Mesenteric Lymph Nodes Both with Host Cells and Autonomously. J. Immunol..

[26] Gonzalez R.J., Lane M.C., Wagner N.J., Weening E.H., Miller V.L. (2015). Dissemination of a highly virulent pathogen: Tracking the early events that define infection. PLoS Pathog..

[27] Bennett I.L., Beeson P.B. (1954). Bacteremia: A consideration of some experimental and clinical aspects. Yale J. Biol. Med..

[28] Shannon J.G., Hasenkrug A.M., Dorward D.W., Nair V., Carmody A.B., Hinnebusch B.J. (2013). Yersinia pestis subverts the dermal neutrophil response in a mouse model of bubonic plague. MBio.

[29] Balmer M.L., Slack E., de Gottardi A., Lawson M.A., Hapfelmeier S., Miele L., Grieco A., Van Vlierberghe H., Fahrner R., Patuto N. (2014). The liver may act as a firewall mediating mutualism between the host and its gut commensal microbiota. Sci. Transl. Med..

[30] Bar-Haim E., Gat O., Markel G., Cohen H., Shafferman A., Velan B. (2008). Interrelationship between dendritic cell trafficking and Francisella tularensis dissemination following airway infection. PLoS Pathog..

[31] Bou Ghanem E.N., Jones G.S., Myers-Morales T., Patil P.D., Hidayatullah A.N., D’Orazio S.E. (2012). InlA promotes dissemination of Listeria monocytogenes to the mesenteric lymph nodes during food borne infection of mice. PLoS Pathog..

[32] St John A.L., Abraham S.N. (2009). Salmonella disrupts lymph node architecture by TLR4-mediated suppression of homeostatic chemokines. Nat. Med..

[33] Chackerian A.A., Alt J.M., Perera T.V., Dascher C.C., Behar S.M. (2002). Dissemination of Mycobacterium tuberculosis is influenced by host factors and precedes the initiation of T-cell immunity. Infect. Immun..

[34] Ganchua S.K.C., Cadena A.M., Maiello P., Gideon H.P., Myers A.J., Junecko B.F., Klein E.C., Lin P.L., Mattila J.T., Flynn J.L. (2018). Lymph nodes are sites of prolonged bacterial persistence during Mycobacterium tuberculosis infection in macaques. PLoS Pathog..

[35] Kastenmuller W., Torabi-Parizi P., Subramanian N., Lammermann T., Germain R.N. (2012). A spatially-organized multicellular innate immune response in lymph nodes limits systemic pathogen spread. Cell.

[36] Bogoslowski A., Butcher E.C., Kubes P. (2018). Neutrophils recruited through high endothelial venules of the lymph nodes via PNAd intercept disseminating Staphylococcus aureus. Proc. Natl. Acad. Sci. USA.

[37] Schulz R.Z., Warren M.F., Drinker C.K. (1938). The Passage of Rabbit Virulent Type Iii Pneumococci from the Respiratory Tract of Rabbits into the Lymphatics and Blood. J. Exp. Med..

[38] Pullinger G.D., Paulin S.M., Charleston B., Watson P.R., Bowen A.J., Dziva F., Morgan E., Villarreal-Ramos B., Wallis T.S., Stevens M.P. (2007). Systemic translocation of Salmonella enterica serovar Dublin in cattle occurs predominantly via efferent lymphatics in a cell-free niche and requires type III secretion system 1 (T3SS-1) but not T3SS-2. Infect. Immun..

[39] Lynskey N.N., Lawrenson R.A., Sriskandan S. (2011). New understandings in *Streptococcus pyogenes*. Curr. Opin. Infect. Dis..

[40] Gonzalez R.J., Miller V.L. (2016). A Deadly Path: Bacterial Spread During Bubonic Plague. Trends Microbiol..

[41] Shannon J.G., Bosio C.F., Hinnebusch B.J. (2015). Dermal neutrophil, macrophage and dendritic cell responses to Yersinia pestis transmitted by fleas. PLoS Pathog..

[42] Sebbane F., Gardner D., Long D., Gowen B.B., Hinnebusch B.J. (2005). Kinetics of disease progression and host response in a rat model of bubonic plague. Am. J. Pathol..

[43] Hughes R., May A.J., Widdicombe J.G. (1956). The role of the lymphatic system in the pathogenesis of anthrax. Br. J. Exp. Pathol..

[44] Reddick L.E., Alto N.M. (2014). Bacteria fighting back: How pathogens target and subvert the host innate immune system. Mol. Cell.

[45] Thammavongsa V., Kim H.K., Missiakas D., Schneewind O. (2015). Staphylococcal manipulation of host immune responses. Nat. Rev. Microbiol..

[46] Angevine D.M. (1936). The Fate of a Virulent Hemolytic Streptococcus Injected into the Skin of Normal and Immunized Rabbits. J. Exp. Med..

[47] Moxon E.R., Kroll J.S. (1990). The role of bacterial polysaccharide capsules as virulence factors. Curr. Top. Microbiol. Immunol..

[48] Kim H.K., Missiakas D., Schneewind O. (2014). Mouse models for infectious diseases caused by Staphylococcus aureus. J. Immunol. Methods.

[49] Weinstein M.P., Towns M.L., Quartey S.M., Mirrett S., Reimer L.G., Parmigiani G., Reller L.B. (1997). The clinical significance of positive blood cultures in the 1990s: A prospective comprehensive evaluation of the microbiology, epidemiology, and outcome of bacteremia and fungemia in adults. Clin. Infect. Dis. Off. Publ. Infect. Dis. Soc. Am..

[50] Pizarro-Cerda J., Cossart P. (2006). Bacterial adhesion and entry into host cells. Cell.

[51] Kim M., Ashida H., Ogawa M., Yoshikawa Y., Mimuro H., Sasakawa C. (2010). Bacterial interactions with the host epithelium. Cell Host Microbe.

[52] Smith S.N., Hagan E.C., Lane M.C., Mobley H.L. (2010). Dissemination and systemic colonization of uropathogenic Escherichia coli in a murine model of bacteremia. MBio.

[53] Hamilton S.M., Bayer C.R., Stevens D.L., Lieber R.L., Bryant A.E. (2008). Muscle injury, vimentin expression, and nonsteroidal anti-inflammatory drugs predispose to cryptic group A streptococcal necrotizing infection. J. Infect. Dis..

[54] Tan L.K.K., Reglinski M., Teo D., Reza N., Lamb L.E.M., Nageshwaran V., Turner C.E., Wikstrom M., Frick I.M., Bjorck L. (2021). Vaccine-induced, but not natural immunity, against the Streptococcal inhibitor of complement protects against invasive disease. npj Vaccines.

[55] Voedisch S., Koenecke C., David S., Herbrand H., Forster R., Rhen M., Pabst O. (2009). Mesenteric lymph nodes confine dendritic cell-mediated dissemination of Salmonella enterica serovar Typhimurium and limit systemic disease in mice. Infect. Immun..

[56] Mellhammar L., Kahn F., Whitlow C., Kander T., Christensson B., Linder A. (2021). Bacteremic sepsis leads to higher mortality when adjusting for confounders with propensity score matching. Sci. Rep..

[57] Triantafyllou E., Gudd C.L., Mawhin M.A., Husbyn H.C., Trovato F.M., Siggins M.K., O’Connor T., Kudo H., Mukherjee S.K., Wendon J.A. (2021). PD-1 blockade improves Kupffer cell bacterial clearance in acute liver injury. J. Clin. Investig..

[58] Macpherson A.J., Heikenwalder M., Ganal-Vonarburg S.C. (2016). The Liver at the Nexus of Host-Microbial Interactions. Cell Host Microbe.

[59] Lewis S.M., Williams A., Eisenbarth S.C. (2019). Structure and function of the immune system in the spleen. Sci. Immunol..

[60] Deniset J.F., Surewaard B.G., Lee W.Y., Kubes P. (2017). Splenic Ly6G(high) mature and Ly6G(int) immature neutrophils contribute to eradication of S. pneumoniae. J. Exp. Med..

[61] Zeng Z., Surewaard B.G., Wong C.H., Geoghegan J.A., Jenne C.N., Kubes P. (2016). CRIg Functions as a Macrophage Pattern Recognition Receptor to Directly Bind and Capture Blood-Borne Gram-Positive Bacteria. Cell Host Microbe.

[62] Lockhart P.B., Brennan M.T., Sasser H.C., Fox P.C., Paster B.J., Bahrani-Mougeot F.K. (2008). Bacteremia associated with toothbrushing and dental extraction. Circulation.

[63] Francois M., Bingen E.H., Lambert-Zechovsky N.Y., Mariani-Kurkdjian P., Nottet J.B., Narcy P. (1992). Bacteremia during tonsillectomy. Arch. Otolaryngol. Head Neck Surg..

[64] Ercoli G., Fernandes V.E., Chung W.Y., Wanford J.J., Thomson S., Bayliss C.D., Straatman K., Crocker P.R., Dennison A., Martinez-Pomares L. (2018). Intracellular replication of Streptococcus pneumoniae inside splenic macrophages serves as a reservoir for septicaemia. Nat. Microbiol..

[65] Holland T.L., Baddour L.M., Bayer A.S., Hoen B., Miro J.M., Fowler V.G. (2016). Infective endocarditis. Nat. Rev. Dis. Primers.

[66] Gaya M., Castello A., Montaner B., Rogers N., Reis e Sousa C., Bruckbauer A., Batista F.D. (2015). Host response. Inflammation-induced disruption of SCS macrophages impairs B cell responses to secondary infection. Science.

[67] Kamenyeva O., Boularan C., Kabat J., Cheung G.Y., Cicala C., Yeh A.J., Chan J.L., Periasamy S., Otto M., Kehrl J.H. (2015). Neutrophil recruitment to lymph nodes limits local humoral response to Staphylococcus aureus. PLoS Pathog..

[68] Cunningham A.F., Gaspal F., Serre K., Mohr E., Henderson I.R., Scott-Tucker A., Kenny S.M., Khan M., Toellner K.M., Lane P.J. (2007). Salmonella induces a switched antibody response without germinal centers that impedes the extracellular spread of infection. J. Immunol..

[69] Sharma H., Turner C.E., Siggins M.K., El-Bahrawy M., Pichon B., Kearns A., Sriskandan S. (2019). Toxic Shock Syndrome Toxin 1 Evaluation and Antibiotic Impact in a Transgenic Model of Staphylococcal Soft Tissue Infection. Msphere.

[70] Griffin A.J., Li L.X., Voedisch S., Pabst O., McSorley S.J. (2011). Dissemination of persistent intestinal bacteria via the mesenteric lymph nodes causes typhoid relapse. Infect. Immun..

[71] Renaud B., Brun-Buisson C., Group I.C.-B.S. (2001). Outcomes of primary and catheter-related bacteremia. A cohort and case-control study in critically ill patients. Am. J. Respir. Crit. Care Med..

[72] Moses A.E., Goldberg S., Korenman Z., Ravins M., Hanski E., Shapiro M. (2002). Invasive group a streptococcal infections, Israel. Emerg. Infect. Dis..

[73] Nelson G.E., Pondo T., Toews K.A., Farley M.M., Lindegren M.L., Lynfield R., Aragon D., Zansky S.M., Watt J.P., Cieslak P.R. (2016). Epidemiology of Invasive Group A Streptococcal Infections in the United States, 2005–2012. Clin. Infect. Dis. Off. Publ. Infect. Dis. Soc. Am..

[74] Lamagni T.L., Darenberg J., Luca-Harari B., Siljander T., Efstratiou A., Henriques-Normark B., Vuopio-Varkila J., Bouvet A., Creti R., Ekelund K. (2008). Epidemiology of severe *Streptococcus pyogenes* disease in Europe. J. Clin. Microbiol..

[75] Adams E.M., Gudmundsson S., Yocum D.E., Haselby R.C., Craig W.A., Sundstrom W.R. (1985). Streptococcal myositis. Arch. Intern. Med..

[76] Stevens D.L., Tanner M.H., Winship J., Swarts R., Ries K.M., Schlievert P.M., Kaplan E. (1989). Severe group A streptococcal infections associated with a toxic shock-like syndrome and scarlet fever toxin A. N. Engl. J. Med..

[77] Stevens D.L., Bryant A.E., Ferretti J.J., Stevens D.L., Fischetti V.A. (2016). Severe Group A Streptococcal Infections. Streptococcus Pyogenes: Basic Biology to Clinical Manifestations.

[78] Prudent E., La Scola B., Drancourt M., Angelakis E., Raoult D. (2018). Molecular strategy for the diagnosis of infectious lymphadenitis. Eur. J. Clin. Microbiol. Infect. Dis..

[79] Wells C.L., Jechorek R.P., Twiggs L.B., Brooker D.C. (1990). Recovery of viable bacteria from pelvic lymph nodes of patients with gynecologic tumors. J. Infect. Dis..

[80] Sakamoto H., Naito H., Ohta Y., Tanakna R., Maeda N., Sasaki J., Nord C.E. (1999). Isolation of bacteria from cervical lymph nodes in patients with oral cancer. Arch. Oral Biol..

[81] Sakamoto H., Sasaki J., Nord C.E. (1999). Association between bacterial colonization on the tumor, bacterial translocation to the cervical lymph nodes and subsequent postoperative infection in patients with oral cancer. Clin. Microbiol. Infect..

[82] O’Boyle C.J., MacFie J., Mitchell C.J., Johnstone D., Sagar P.M., Sedman P.C. (1998). Microbiology of bacterial translocation in humans. Gut.

[83] Stevens D.L., Bryant A.E., Ferretti J.J., Stevens D.L., Fischetti V.A. (2016). Impetigo, Erysipelas and Cellulitis. Streptococcus Pyogenes: Basic Biology to Clinical Manifestations.

[84] Stevens D.L., Bryant A.E. (2017). Necrotizing Soft-Tissue Infections. N. Engl. J. Med..

[85] Ackermann M., Stecher B., Freed N.E., Songhet P., Hardt W.D., Doebeli M. (2008). Self-destructive cooperation mediated by phenotypic noise. Nature.

[86] Cywes C., Wessels M.R. (2001). Group A Streptococcus tissue invasion by CD44-mediated cell signalling. Nature.

[87] Takeuchi O., Akira S. (2010). Pattern recognition receptors and inflammation. Cell.

[88] Moore J.E., Bertram C.D. (2018). Lymphatic System Flows. Annu. Rev. Fluid Mech..

[89] Polomska A.K., Proulx S.T. (2021). Imaging technology of the lymphatic system. Adv. Drug Deliv. Rev..

[90] Sarin H. (2010). Physiologic upper limits of pore size of different blood capillary types and another perspective on the dual pore theory of microvascular permeability. J. Angiogenesis Res..

[91] Lea A.W. (1905). Some Remarks on Puerperal Infection. Br. Med. J..

[92] Taylor J. (1931). Puerperal Infection. Postgrad. Med. J..

[93] Baluk P., Fuxe J., Hashizume H., Romano T., Lashnits E., Butz S., Vestweber D., Corada M., Molendini C., Dejana E. (2007). Functionally specialized junctions between endothelial cells of lymphatic vessels. J. Exp. Med..

[94] Leak L.V., Burke J.F. (1968). Ultrastructural studies on the lymphatic anchoring filaments. J. Cell Biol..

[95] Ikomi F., Hunt J., Hanna G., Schmid-Schonbein G.W. (1996). Interstitial fluid, plasma protein, colloid, and leukocyte uptake into initial lymphatics. J. Appl. Physiol..

[96] Trzewik J., Mallipattu S.K., Artmann G.M., Delano F.A., Schmid-Schonbein G.W. (2001). Evidence for a second valve system in lymphatics: Endothelial microvalves. FASEB J..

[97] Johnson L.A., Banerji S., Lawrance W., Gileadi U., Prota G., Holder K.A., Roshorm Y.M., Hanke T., Cerundolo V., Gale N.W. (2017). Dendritic cells enter lymph vessels by hyaluronan-mediated docking to the endothelial receptor LYVE-1. Nat. Immunol..

[98] Jamalian S., Jafarnejad M., Zawieja S.D., Bertram C.D., Gashev A.A., Zawieja D.C., Davis M.J., Moore J.E. (2017). Demonstration and Analysis of the Suction Effect for Pumping Lymph from Tissue Beds at Subatmospheric Pressure. Sci. Rep..

[99] Jackson D.G. (2014). Lymphatic Regulation of Cellular Trafficking. J. Clin. Cell Immunol.

[100] Jackson D.G. (2019). Leucocyte Trafficking via the Lymphatic Vasculature- Mechanisms and Consequences. Front. Immunol..

[101] Oh S.Y., Budzik J.M., Garufi G., Schneewind O. (2011). Two capsular polysaccharides enable Bacillus cereus G9241 to cause anthrax-like disease. Mol. Microbiol..

[102] Guss B., Flock M., Frykberg L., Waller A.S., Robinson C., Smith K.C., Flock J.I. (2009). Getting to grips with strangles: An effective multi-component recombinant vaccine for the protection of horses from Streptococcus equi infection. PLoS Pathog..

[103] Harper M., Boyce J.D., Adler B. (2006). Pasteurella multocida pathogenesis: 125 years after Pasteur. FEMS Microbiol. Lett..

[104] McMaster P.D., Hudack S.S. (1934). The Participation of Skin Lymphatics in Repair of the Lesions Due to Incisions and Burns. J. Exp. Med..

[105] Barnes J.M., Trueta J. (1941). Absorption of Bacteria, Toxins and Snake Venoms from the Tissues: Importance of the Lymphatic Circulation. Lancet.

[106] Claesson-Welsh L., Dejana E., McDonald D.M. (2021). Permeability of the Endothelial Barrier: Identifying and Reconciling Controversies. Trends Mol. Med..

[107] McDonald D.M., Thurston G., Baluk P. (1999). Endothelial gaps as sites for plasma leakage in inflammation. Microcirculation.

[108] Miteva D.O., Rutkowski J.M., Dixon J.B., Kilarski W., Shields J.D., Swartz M.A. (2010). Transmural flow modulates cell and fluid transport functions of lymphatic endothelium. Circ. Res..

[109] Baluk P., Hirata A., Thurston G., Fujiwara T., Neal C.R., Michel C.C., McDonald D.M. (1997). Endothelial gaps: Time course of formation and closure in inflamed venules of rats. Am. J. Physiol..

[110] Baffert F., Le T., Thurston G., McDonald D.M. (2006). Angiopoietin-1 decreases plasma leakage by reducing number and size of endothelial gaps in venules. Am. J. Physiol. Heart Circ. Physiol..

[111] Aldrich M.B., Sevick-Muraca E.M. (2013). Cytokines are systemic effectors of lymphatic function in acute inflammation. Cytokine.

[112] Cromer W.E., Zawieja S.D., Tharakan B., Childs E.W., Newell M.K., Zawieja D.C. (2014). The effects of inflammatory cytokines on lymphatic endothelial barrier function. Angiogenesis.

[113] Chary S.R., Jain R.K. (1989). Direct measurement of interstitial convection and diffusion of albumin in normal and neoplastic tissues by fluorescence photobleaching. Proc. Natl. Acad. Sci. USA.

[114] He C., Young A.J., West C.A., Su M., Konerding M.A., Mentzer S.J. (2002). Stimulation of regional lymphatic and blood flow by epicutaneous oxazolone. J. Appl. Physiol..

[115] Bohlen H.G., Wang W., Gashev A., Gasheva O., Zawieja D. (2009). Phasic contractions of rat mesenteric lymphatics increase basal and phasic nitric oxide generation in vivo. Am. J. Physiol. Heart Circ. Physiol..

[116] Lamb L.E., Siggins M.K., Scudamore C., Macdonald W., Turner C.E., Lynskey N.N., Tan L.K.K., Sriskandan S. (2018). Impact of contusion injury on intramuscular emm1 group a streptococcus infection and lymphatic spread. Virulence.

[117] Swartz M.A., Berk D.A., Jain R.K. (1996). Transport in lymphatic capillaries. I. Macroscopic measurements using residence time distribution theory. Am. J. Physiol..

[118] Reddy S.T., van der Vlies A.J., Simeoni E., Angeli V., Randolph G.J., O’Neil C.P., Lee L.K., Swartz M.A., Hubbell J.A. (2007). Exploiting lymphatic transport and complement activation in nanoparticle vaccines. Nat. Biotechnol..

[119] Manolova V., Flace A., Bauer M., Schwarz K., Saudan P., Bachmann M.F. (2008). Nanoparticles target distinct dendritic cell populations according to their size. Eur. J. Immunol..

[120] Trevaskis N.L., Kaminskas L.M., Porter C.J. (2015). From sewer to saviour-targeting the lymphatic system to promote drug exposure and activity. Nat. Rev. Drug Discov..

[121] Supersaxo A., Hein W.R., Steffen H. (1990). Effect of molecular weight on the lymphatic absorption of water-soluble compounds following subcutaneous administration. Pharm. Res..

[122] Abel S., Abel zur Wiesch P., Davis B.M., Waldor M.K. (2015). Analysis of Bottlenecks in Experimental Models of Infection. PLoS Pathog..

[123] Lim C.H., Voedisch S., Wahl B., Rouf S.F., Geffers R., Rhen M., Pabst O. (2014). Independent bottlenecks characterize colonization of systemic compartments and gut lymphoid tissue by salmonella. PLoS Pathog..

[124] Kono M., Zafar M.A., Zuniga M., Roche A.M., Hamaguchi S., Weiser J.N. (2016). Single Cell Bottlenecks in the Pathogenesis of Streptococcus pneumoniae. PLoS Pathog..

[125] Moxon E.R., Murphy P.A. (1978). Haemophilus influenzae bacteremia and meningitis resulting from survival of a single organism. Proc. Natl. Acad. Sci. USA.

[126] Theocharis A.D., Skandalis S.S., Gialeli C., Karamanos N.K. (2016). Extracellular matrix structure. Adv. Drug Deliv. Rev..

[127] Mouw J.K., Ou G., Weaver V.M. (2014). Extracellular matrix assembly: A multiscale deconstruction. Nat. Rev. Mol. Cell Biol..

[128] Frantz C., Stewart K.M., Weaver V.M. (2010). The extracellular matrix at a glance. J. Cell Sci..

[129] Bachmann M.F., Jennings G.T. (2010). Vaccine delivery: A matter of size, geometry, kinetics and molecular patterns. Nat. Rev. Immunol..

[130] Porter C.J. (1997). Drug delivery to the lymphatic system. Crit. Rev. Drug Carr. Syst..

[131] Comper W.D., Laurent T.C. (1978). Physiological function of connective tissue polysaccharides. Physiol. Rev..

[132] Wiig H., Swartz M.A. (2012). Interstitial fluid and lymph formation and transport: Physiological regulation and roles in inflammation and cancer. Physiol. Rev..

[133] Swartz M.A. (2001). The physiology of the lymphatic system. Adv. Drug Deliv. Rev..

[134] Rao D.A., Forrest M.L., Alani A.W., Kwon G.S., Robinson J.R. (2010). Biodegradable PLGA based nanoparticles for sustained regional lymphatic drug delivery. J. Pharm. Sci..

[135] Bagby T.R., Duan S., Cai S., Yang Q., Thati S., Berkland C., Aires D.J., Forrest M.L. (2012). Lymphatic trafficking kinetics and near-infrared imaging using star polymer architectures with controlled anionic character. Eur. J. Pharm. Sci..

[136] Tomlin H., Piccinini A.M. (2018). A complex interplay between the extracellular matrix and the innate immune response to microbial pathogens. Immunology.

[137] Sorokin L. (2010). The impact of the extracellular matrix on inflammation. Nat. Rev. Immunol..

[138] Winkler J., Abisoye-Ogunniyan A., Metcalf K.J., Werb Z. (2020). Concepts of extracellular matrix remodelling in tumour progression and metastasis. Nat. Commun..

[139] Schembri M.A., Dalsgaard D., Klemm P. (2004). Capsule shields the function of short bacterial adhesins. J. Bacteriol..

[140] Hollands A., Pence M.A., Timmer A.M., Osvath S.R., Turnbull L., Whitchurch C.B., Walker M.J., Nizet V. (2010). Genetic switch to hypervirulence reduces colonization phenotypes of the globally disseminated group A streptococcus M1T1 clone. J. Infect. Dis..

[141] Absolom D.R. (1988). The role of bacterial hydrophobicity in infection: Bacterial adhesion and phagocytic ingestion. Can. J. Microbiol..

[142] Fletcher M., Loeb G.I. (1979). Influence of substratum characteristics on the attachment of a marine pseudomonad to solid surfaces. Appl. Environ. Microbiol..

[143] Kuusela P. (1978). Fibronectin binds to Staphylococcus aureus. Nature.

[144] Heilmann C. (2011). Adhesion mechanisms of staphylococci. Adv. Exp. Med. Biol..

[145] Siggins M.K., Gill S.K., Langford P.R., Li Y., Ladhani S.N., Tregoning J.S. (2015). PHiD-CV induces anti-Protein D antibodies but does not augment pulmonary clearance of nontypeable Haemophilus influenzae in mice. Vaccine.

[146] Gilbert P., Evans D.J., Evans E., Duguid I.G., Brown M.R. (1991). Surface characteristics and adhesion of Escherichia coli and Staphylococcus epidermidis. J. Appl. Bacteriol..

[147] Gryllos I., Cywes C., Shearer M.H., Cary M., Kennedy R.C., Wessels M.R. (2001). Regulation of capsule gene expression by group A Streptococcus during pharyngeal colonization and invasive infection. Mol. Microbiol..

[148] Schmid-Schonbein G.W. (1990). Microlymphatics and lymph flow. Physiol. Rev..

[149] Kuan E.L., Ivanov S., Bridenbaugh E.A., Victora G., Wang W., Childs E.W., Platt A.M., Jakubzick C.V., Mason R.J., Gashev A.A. (2015). Collecting lymphatic vessel permeability facilitates adipose tissue inflammation and distribution of antigen to lymph node-homing adipose tissue dendritic cells. J. Immunol..

[150] Margaris K.N., Black R.A. (2012). Modelling the lymphatic system: Challenges and opportunities. J. R. Soc. Interface.

[151] Willard-Mack C.L. (2006). Normal structure, function, and histology of lymph nodes. Toxicol. Pathol..

[152] Van den Broeck W., Derore A., Simoens P. (2006). Anatomy and nomenclature of murine lymph nodes: Descriptive study and nomenclatory standardization in BALB/cAnNCrl mice. J. Immunol. Methods.

[153] Nossal G.J., Abbot A., Mitchell J. (1968). Antigens in immunity. XIV. Electron microscopic radioautographic studies of antigen capture in the lymph node medulla. J. Exp. Med..

[154] Foley M.J., Smith M.R., Wood W.B. (1959). Studies on the pathogenicity of group A Streptococci. I. Its relation to surface phagocytosis. J. Exp. Med..

[155] Field M.E., Drinker C.K., White J.C. (1932). Lymph Pressures in Sterile Inflammation. J. Exp. Med..

[156] Benoit J.N., Zawieja D.C. (1992). Effects of f-Met-Leu-Phe-induced inflammation on intestinal lymph flow and lymphatic pump behavior. Am. J. Physiol..

[157] Fossum S. (1980). The architecture of rat lymph nodes. IV. Distribution of ferritin and colloidal carbon in the draining lymph nodes after foot-pad injection. Scand. J. Immunol..

[158] Rantakari P., Auvinen K., Jappinen N., Kapraali M., Valtonen J., Karikoski M., Gerke H., Iftakhar E.K.I., Keuschnigg J., Umemoto E. (2015). The endothelial protein PLVAP in lymphatics controls the entry of lymphocytes and antigens into lymph nodes. Nat. Immunol..

[159] Sixt M., Kanazawa N., Selg M., Samson T., Roos G., Reinhardt D.P., Pabst R., Lutz M.B., Sorokin L. (2005). The conduit system transports soluble antigens from the afferent lymph to resident dendritic cells in the T cell area of the lymph node. Immunity.

[160] Siggins M.K., Thwaites R.S., Openshaw P.J.M. (2021). Durability of Immunity to SARS-CoV-2 and Other Respiratory Viruses. Trends Microbiol..

[161] Liao S., von der Weid P.Y. (2015). Lymphatic system: An active pathway for immune protection. Semin. Cell Dev. Biol..

[162] Smith M.R., Wood W.B. (1958). Surface phagocy.ytosis; further evidence of its destructive action upon fully encapsulated pneumococci in the absence of type-specific antibody. J. Exp. Med..

[163] Smith R.O., Wood W.B. (1949). Cellular mechanisms of antibacterial defense in lymph nodes; pathogenesis of acute bacterial lymphadenitis. J. Exp. Med..

[164] Sansonetti P. (2001). Phagocytosis of bacterial pathogens: Implications in the host response. Semin. Immunol..

[165] Garcia Z., Lemaitre F., van Rooijen N., Albert M.L., Levy Y., Schwartz O., Bousso P. (2012). Subcapsular sinus macrophages promote NK cell accumulation and activation in response to lymph-borne viral particles. Blood.

[166] Moseman E.A., Iannacone M., Bosurgi L., Tonti E., Chevrier N., Tumanov A., Fu Y.X., Hacohen N., von Andrian U.H. (2012). B cell maintenance of subcapsular sinus macrophages protects against a fatal viral infection independent of adaptive immunity. Immunity.

[167] Takeda A., Hollmen M., Dermadi D., Pan J., Brulois K.F., Kaukonen R., Lonnberg T., Bostrom P., Koskivuo I., Irjala H. (2019). Single-Cell Survey of Human Lymphatics Unveils Marked Endothelial Cell Heterogeneity and Mechanisms of Homing for Neutrophils. Immunity.

[168] Xiang M., Grosso R.A., Takeda A., Pan J., Bekkhus T., Brulois K., Dermadi D., Nordling S., Vanlandewijck M., Jalkanen S. (2020). A Single-Cell Transcriptional Roadmap of the Mouse and Human Lymph Node Lymphatic Vasculature. Front. Cardiovasc. Med..

[169] Jalkanen S., Salmi M. (2020). Lymphatic endothelial cells of the lymph node. Nat. Rev. Immunol..

[170] Jafarnejad M., Woodruff M.C., Zawieja D.C., Carroll M.C., Moore J.E. (2015). Modeling Lymph Flow and Fluid Exchange with Blood Vessels in Lymph Nodes. Lymphat. Res. Biol..

[171] Widdicombe J.G., Hughes R., May A.J. (1955). The efficiency of filtration by the popliteal lymph node of the rabbit. Br. J. Exp. Pathol..

[172] Bogoslowski A., Wijeyesinghe S., Lee W.Y., Chen C.S., Alanani S., Jenne C., Steeber D.A., Scheiermann C., Butcher E.C., Masopust D. (2020). Neutrophils Recirculate through Lymph Nodes to Survey Tissues for Pathogens. J. Immunol..

[173] Chtanova T., Schaeffer M., Han S.J., van Dooren G.G., Nollmann M., Herzmark P., Chan S.W., Satija H., Camfield K., Aaron H. (2008). Dynamics of neutrophil migration in lymph nodes during infection. Immunity.

[174] Hampton H.R., Bailey J., Tomura M., Brink R., Chtanova T. (2015). Microbe-dependent lymphatic migration of neutrophils modulates lymphocyte proliferation in lymph nodes. Nat. Commun..

[175] Rigby D.A., Ferguson D.J., Johnson L.A., Jackson D.G. (2015). Neutrophils rapidly transit inflamed lymphatic vessel endothelium via integrin-dependent proteolysis and lipoxin-induced junctional retraction. J. Leukoc. Biol..

[176] Phan T.G., Green J.A., Gray E.E., Xu Y., Cyster J.G. (2009). Immune complex relay by subcapsular sinus macrophages and noncognate B cells drives antibody affinity maturation. Nat. Immunol..

[177] Cyster J.G. (2010). B cell follicles and antigen encounters of the third kind. Nat. Immunol..

[178] Moran I., Grootveld A.K., Nguyen A., Phan T.G. (2018). Subcapsular Sinus Macrophages: The Seat of Innate and Adaptive Memory in Murine Lymph Nodes. Trends Immunol..

[179] Carrasco Y.R., Batista F.D. (2007). B cells acquire particulate antigen in a macrophage-rich area at the boundary between the follicle and the subcapsular sinus of the lymph node. Immunity.

[180] Farrell H.E., Davis-Poynter N., Bruce K., Lawler C., Dolken L., Mach M., Stevenson P.G. (2015). Lymph Node Macrophages Restrict Murine Cytomegalovirus Dissemination. J. Virol..

[181] Chang Y.C., Olson J., Louie A., Crocker P.R., Varki A., Nizet V. (2014). Role of macrophage sialoadhesin in host defense against the sialylated pathogen group B Streptococcus. J. Mol. Med. (Berl.).

[182] Heikema A.P., Koning R.I., Duarte dos Santos Rico S., Rempel H., Jacobs B.C., Endtz H.P., van Wamel W.J., Samsom J.N. (2013). Enhanced, sialoadhesin-dependent uptake of Guillain-Barre syndrome-associated Campylobacter jejuni strains by human macrophages. Infect. Immun..

[183] Jones C., Virji M., Crocker P.R. (2003). Recognition of sialylated meningococcal lipopolysaccharide by siglecs expressed on myeloid cells leads to enhanced bacterial uptake. Mol. Microbiol..

[184] Phan T.G., Grigorova I., Okada T., Cyster J.G. (2007). Subcapsular encounter and complement-dependent transport of immune complexes by lymph node B cells. Nat. Immunol..

[185] Szakal A.K., Holmes K.L., Tew J.G. (1983). Transport of immune complexes from the subcapsular sinus to lymph node follicles on the surface of nonphagocytic cells, including cells with dendritic morphology. J. Immunol..

[186] Gonzalez S.F., Lukacs-Kornek V., Kuligowski M.P., Pitcher L.A., Degn S.E., Kim Y.A., Cloninger M.J., Martinez-Pomares L., Gordon S., Turley S.J. (2010). Capture of influenza by medullary dendritic cells via SIGN-R1 is essential for humoral immunity in draining lymph nodes. Nat. Immunol..

[187] Lynskey N.N., Reglinski M., Calay D., Siggins M.K., Mason J.C., Botto M., Sriskandan S. (2017). Multi-functional mechanisms of immune evasion by the streptococcal complement inhibitor C5a peptidase. PLoS Pathog..

[188] Rooijakkers S.H., Ruyken M., Roos A., Daha M.R., Presanis J.S., Sim R.B., van Wamel W.J., van Kessel K.P., van Strijp J.A. (2005). Immune evasion by a staphylococcal complement inhibitor that acts on C3 convertases. Nat. Immunol..

[189] Lambris J.D., Ricklin D., Geisbrecht B.V. (2008). Complement evasion by human pathogens. Nat. Rev. Microbiol..

[190] Geijtenbeek T.B., Groot P.C., Nolte M.A., van Vliet S.J., Gangaram-Panday S.T., van Duijnhoven G.C., Kraal G., van Oosterhout A.J., van Kooyk Y. (2002). Marginal zone macrophages express a murine homologue of DC-SIGN that captures blood-borne antigens in vivo. Blood.

[191] Martens J.H., Kzhyshkowska J., Falkowski-Hansen M., Schledzewski K., Gratchev A., Mansmann U., Schmuttermaier C., Dippel E., Koenen W., Riedel F. (2006). Differential expression of a gene signature for scavenger/lectin receptors by endothelial cells and macrophages in human lymph node sinuses, the primary sites of regional metastasis. J. Pathol..

[192] Lanoue A., Clatworthy M.R., Smith P., Green S., Townsend M.J., Jolin H.E., Smith K.G., Fallon P.G., McKenzie A.N. (2004). SIGN-R1 contributes to protection against lethal pneumococcal infection in mice. J. Exp. Med..

[193] Kang Y.S., Kim J.Y., Bruening S.A., Pack M., Charalambous A., Pritsker A., Moran T.M., Loeffler J.M., Steinman R.M., Park C.G. (2004). The C-type lectin SIGN-R1 mediates uptake of the capsular polysaccharide of Streptococcus pneumoniae in the marginal zone of mouse spleen. Proc. Natl. Acad. Sci. USA.

[194] Van der Laan L.J., Döpp E.A., Haworth R., Pikkarainen T., Kangas M., Elomaa O., Dijkstra C.D., Gordon S., Tryggvason K., Kraal G. (1999). Regulation and functional involvement of macrophage scavenger receptor MARCO in clearance of bacteria in vivo. J. Immunol..

[195] Doyle S.E., O’Connell R.M., Miranda G.A., Vaidya S.A., Chow E.K., Liu P.T., Suzuki S., Suzuki N., Modlin R.L., Yeh W.C. (2004). Toll-like receptors induce a phagocytic gene program through p38. J. Exp. Med..

[196] Loof T.G., Rohde M., Chhatwal G.S., Jung S., Medina E. (2007). The contribution of dendritic cells to host defenses against *Streptococcus pyogenes*. J. Infect. Dis..

[197] Ross J.M. (1957). The pathogenesis of anthrax following the administration of spores by the respiratory route. J. Pathol. Bacteriol..

[198] Angevine D.M. (1934). The Fate of Avirulent Hemolytic Streptococci Injected into the Skin of Normal and Sensitized Rabbits: Local Fixation of Bacteria. J. Exp. Med..

[199] Wilson A.T., Wiley G.G., Bruno P. (1957). Fate of non-virulent group A streptococci phagocytized by human and mouse neutrophils. J. Exp. Med..

[200] Segal A.W. (2005). How neutrophils kill microbes. Annu. Rev. Immunol..

[201] Wilson A.T. (1953). The egestion of phagocytized particles by leukocytes. J. Exp. Med..

[202] St Sauver J.L., Weaver A.L., Orvidas L.J., Jacobson R.M., Jacobsen S.J. (2006). Population-based prevalence of repeated group A beta-hemolytic streptococcal pharyngitis episodes. Mayo Clin. Proc..

[203] Davies F.J., Olme C., Lynskey N.N., Turner C.E., Sriskandan S. (2019). Streptococcal superantigen-induced expansion of human tonsil T cells leads to altered T follicular helper cell phenotype, B cell death and reduced immunoglobulin release. Clin. Exp. Immunol..

[204] Dan J.M., Havenar-Daughton C., Kendric K., Al-Kolla R., Kaushik K., Rosales S.L., Anderson E.L., LaRock C.N., Vijayanand P., Seumois G. (2019). Recurrent group A Streptococcus tonsillitis is an immunosusceptibility disease involving antibody deficiency and aberrant TFH cells. Sci. Transl. Med..

[205] Stollerman G.H., Dale J.B. (2008). The importance of the group a streptococcus capsule in the pathogenesis of human infections: A historical perspective. Clin. Infect. Dis..

[206] Mestas J., Hughes C.C. (2004). Of mice and not men: Differences between mouse and human immunology. J. Immunol..

[207] Siggins M.K., Cunningham A.F., Marshall J.L., Chamberlain J.L., Henderson I.R., MacLennan C.A. (2011). Absent bactericidal activity of mouse serum against invasive African nontyphoidal Salmonella results from impaired complement function but not a lack of antibody. J. Immunol..

[208] Warren H.S., Fitting C., Hoff E., Adib-Conquy M., Beasley-Topliffe L., Tesini B., Liang X., Valentine C., Hellman J., Hayden D. (2010). Resilience to bacterial infection: Difference between species could be due to proteins in serum. J. Infect. Dis..

[209] Hawash M.B.F., Sanz-Remon J., Grenier J.C., Kohn J., Yotova V., Johnson Z., Lanford R.E., Brinkworth J.F., Barreiro L.B. (2021). Primate innate immune responses to bacterial and viral pathogens reveals an evolutionary trade-off between strength and specificity. Proc. Natl. Acad. Sci. USA.

